# Gender-related differentially expressed genes in pancreatic cancer: possible culprits or accomplices?

**DOI:** 10.3389/fgene.2022.966941

**Published:** 2022-10-26

**Authors:** Roya Ramezankhani, Afshin Abdi Ghavidel, Saadyeh Rashidi, Mahbubeh Rojhannezhad, Hamid Reza Abolkheir, Malihe Mirhosseini, Sara Taleahmad, Massoud Vosough

**Affiliations:** ^1^ Department of Development and Regeneration, Stem Cell Biology and Embryology, KU Leuven Stem Cell Institute, Leuven, Belgium; ^2^ Department of Applied Cell Sciences, Faculty of Basic Science and Advanced Medical Technologies, Royan Institute, ACECR, Tehran, Iran; ^3^ Student Research Committee, School of Advanced Technologies in Medicine, Shahid Beheshti University of Medical Sciences, Tehran, Iran; ^4^ Department of Biology, Faculty of Biological Sciences and Technology, Shahid Beheshti University, Tehran, Iran; ^5^ Department of Genetics, Faculty of Biological Sciences, Tarbiat Modares University, Tehran, Iran; ^6^ School of Biomedical Engineering, University of Technology Sydney, Sydney, NSW, Australia; ^7^ Department of Stem Cells and Developmental Biology, Cell Science Research Center, Royan Institute for Stem Cell Biology and Technology, ACECR, Tehran, Iran; ^8^ Department of Regenerative Medicine, Cell Science Research Center, Royan Institute for Stem Cell Biology and Technology, Academic Center for Education, Culture and Research, ACECR, Tehran, Iran; ^9^ Experimental Cancer Medicine, Institution for Laboratory Medicine, Karolinska Institute, Stockholm, Sweden

**Keywords:** pancreatic cancer, gender propensity, extracellular matrix, androgen receptor, estrogen receptor

## Abstract

Pancreatic cancer (PC) is one of the leading causes of cancer mortality worldwide, and its incidence and mortality rate in several regions is higher in male patients. Although numerous efforts have been made to enhance the clinical outcomes of existing therapeutic regimens, their efficiency is still low, and drug resistance usually occurs in many patients. In addition, the exact underlying molecular basis that makes PC slightly more prevalent among males remains unknown. Providing information regarding the possible association between gender and PC tumorigenesis may offer important clues for how certain molecular cross-talks can affect PC initiation and/or progression. In this study, we used several microarray expression data to identify the common up- and downregulated genes within one specific gender, which were also specified to have binding sites for androgen and/or estrogen receptors. Using functional enrichment analysis among the others, for all the gene sets found in this study, we have shed light on the plausible importance of the androgenic effectors in tumorigenesis, such as the androgen-regulated expression of the GLI transcription factor and the potential role of testosterone in the extracellular matrix (ECM)–cell interaction, which are known for their importance in tumorigenesis. Moreover, we demonstrated that the biological process *axon guidance* was highlighted regarding the upregulated genes in male patients. Overall, identification of gene candidates as the possible link between gender and PC progression or survival rates may help in developing strategies to reduce the incidence of this cancer.

## 1 Introduction

Pancreatic cancer (PC) is the seventh leading cause of cancer deaths worldwide, and it is expected that its rank would even go higher (Rawla et al., 2019; Hu et al., 2021). Despite significant improvements in the overall outcome of cancer patients, the incidence and mortality rates of PC have not showed a substantial change over the past 30 years, and the 5-year survival rate is only about nine percent (Hu et al., 2021). This is due to the late diagnosis and lack of knowledge regarding the major molecular mechanisms of PC tumorigenesis, which have resulted in poor effectiveness of therapeutic approaches and drug resistance (Zhu et al., 2020; Javadrashid et al., 2021). However, one important consideration in PC research may be the influence of gender on the incidence and survival rates of this cancer (Pourshams et al., 2019; Sung et al., 2021). The relatively higher incidence rates of PC in males have been shown in certain regions, for example, in 2020 in Eastern and Western Europe (with an age-standardized incidence rate per 100,000 of 9.9 for males and 5.6 for females and 9.8 for males and 7.4 for females, respectively), Northern America, Southern Europe, and Northern Europe, among others (Sung et al., 2021). One possible reason, aside from females’ less exposure to PC risk factors (Mario et al., 2018) including the growing rates of obesity, diabetes, and alcohol consumption, might be the sex effect on PC development and progression both in humans and rodent animal models (Longnecker and Sumi, 1990; Wang et al., 2021). In fact, glucocorticoids and hormones have shown to affect the physiology of pancreas. Accordingly, in the PC rat models which were treated with azaserine, estrogens could play a protective role, while androgens possibly facilitate the development of PC (Wang et al., 2021). In line with that, evidence also points to the existence of an association between PC and sex hormone signaling in humans. In this regards, several anti-androgen drugs were tested in PC models, and their potential antitumor effect was demonstrated (Schweizer and Yu, 2017; Wang et al., 2021). However, the results of the related clinical trials have shown conflicting results (Schweizer and Yu, 2017).

Histologically, PC is divided into two main types: exocrine tumors (−93%), that is, cystic tumors, acinar cells cancers, and adenocarcinomas, such as pancreatic ductal adenocarcinoma (PDAC), which is the most common type of PC, and endocrine tumors (−7%) (Mostafa et al., 2017). Although various studies have focused on elucidating the underlying molecular mechanisms of the disease, a lot remained to be discovered to help with lowering the mortality rate of this cancer (Rawla et al., 2019; Hu et al., 2021; Javadrashid et al., 2021). PC, as a multifactorial disease, is associated with both genetic and environmental risk factors. Genetic alternations, for example, in *KRAS*, *TP53*, *CDKN2A*, and *SMAD4,2* (as the main drivers of PC), appearance of numerous differentially up- and downregulated genes, and downstream affected cellular signaling pathways have long been shown to be involved in PC progression (Jones et al., 2008; Javadrashid et al., 2021). Transcription factors (TFs) are among the final effectors of these pathways as well as certain hormones (such as androgen and estrogen) and play important roles in cellular processes (Bhagwat and Vakoc, 2015; Naqvi et al., 2018; Islam et al., 2021). Examples of such TFs are androgen receptors (ARs) and estrogen receptors (ERs). Activation of GLI transcription factors, which are involved in PDA oncogenesis in pancreatic epithelium,for example, can be mediated through AR binding (Adams et al., 2019; Li et al., 2018a). In accordance, one novel and interesting function of GLI2 is being the master regulator of the basal-like subset of PDA, which is characterized by the expression of laminin and epithelial-to-mesenchymal transition (EMT) markers, among others (Adams et al., 2019). However, it should be also noted that, for example, in breast cancer cells, non-canonical induction of *GLI3* transcription is driven by an ER interaction (Massah et al., 2021). In addition to the cascade of deregulated pathways with intracellular origins, tumor microenvironment (TME) has also a significant impact on PC progression. This, along with the aberrant cell-ECM homeostasis, is among the leaders of cancer progression, including metastasis and drug resistance (Henke et al., 2020). Moreover, the collagen density has been shown to have a possible role critical for cancer cells to evade immune responses (Kuczek et al., 2019). Fibronectin, another important component of ECM, has also a prominent role in PDAC cell malignancy and fibrogenesis (Javadrashid et al., 2021). One notable consideration in this regards is the possible interaction of physiological testosterone in enhancement of ECM synthesis within certain cells (Bertolo et al., 2014). However, the exact molecular mechanism in PC male patients still needs to be elucidated.

Through specifying differentially expressed gene (DEG) sets in affected versus normal cells and gene expression profiling, bioinformatics analysis has thus far demonstrated its potential to further enlighten the molecular mechanisms involved in PC (Jiang et al., 2015; Wang et al., 2017; Li et al., 2018b). DEGs in certain conditions, such as cancer, may differ among males and females due to their chromosomal locations or their response to the level of steroid hormones, which affect both immunological (innate and adaptive) and non-immunological responses to tumors (Libert et al., 2010; Zhang et al., 2012; Fuentes and Silveyra, 2019; Blencowe et al., 2022). This would lead to the high incidence of autoimmune diseases in females, whereas males show a higher risk of death from cancers (Klein and Flanagan, 2016). Therefore, in this study, we aimed to analyze the likely impact of gender on DEGs in PC and to propose possible candidates in this regards. However, one main drawback of this work was the limited access to the clinical characteristics of patients, which makes the interpretation of clinical relevance in regards with the identified genes difficult. Nevertheless, the suggested perspective here is applicable in various cancer studies and can offer additional benefits. Finding the involved gene(s) and mechanism(s) through which gender would influence PC progression or survival rates may help in finding solutions to improve the effectiveness of therapies and ameliorating the disease progression in PC patients.

## 2 Methods

**FIGURE 1:**
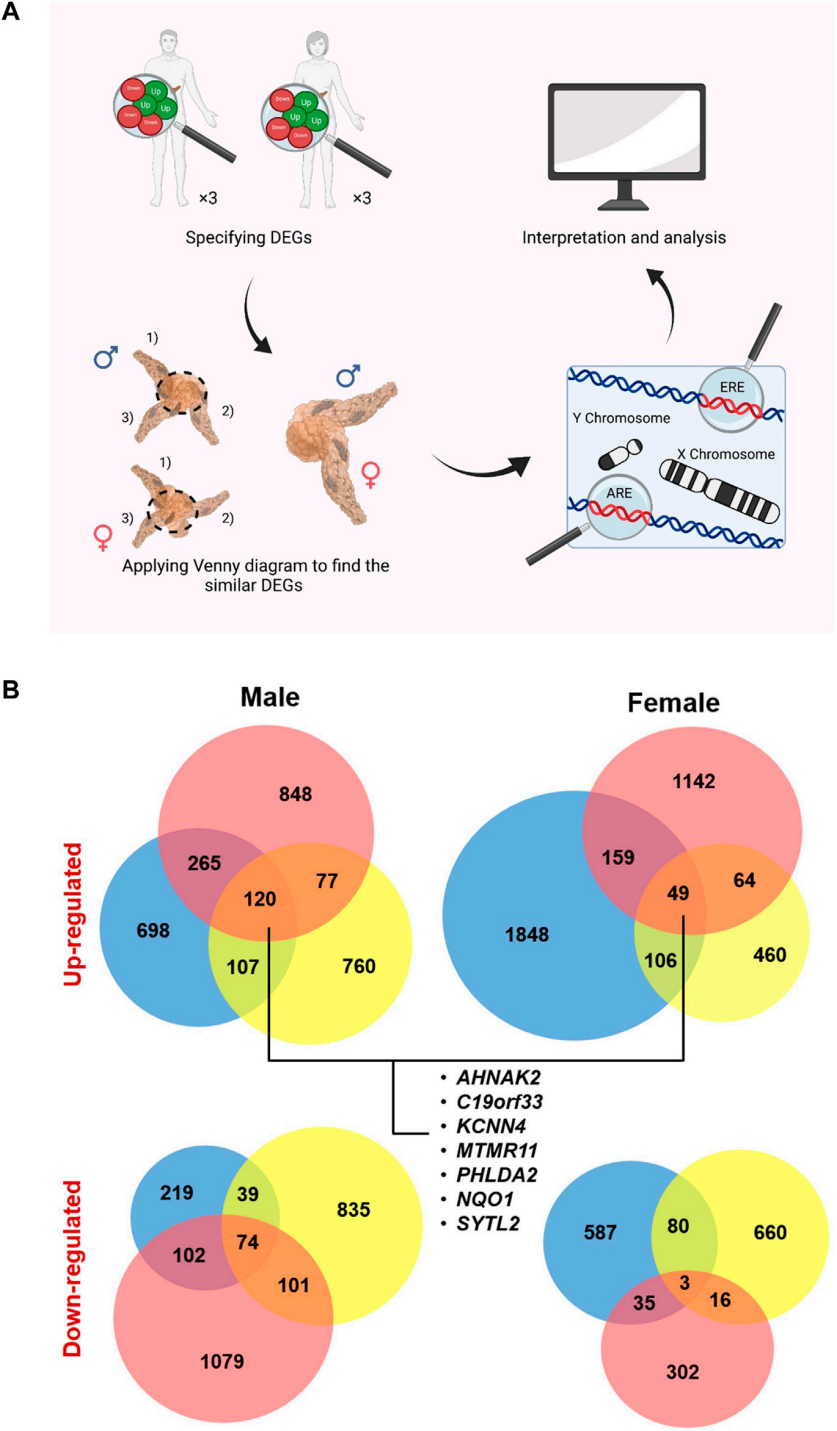
Study design and specified up- and down-regulated genes (U/DRGs). **(A)** Micro array studies for investigating the altered gene expression in both male and female pancreatic cancer (PC) patients were selected and the similar DEGs in both genders or the sex specified DEGs were identified. Subsequently, genes with AREs or EREs, through performing functional enrichment analysis, and the ones located on sex chromosomes were specified. **(B)** The number of identified U/DRGs in both genders. Log FC value ≥1 or ≤ −1 was chosen as the cut-off criterion for the up- and down-regulated genes, respectively, using a p-value of ≤ 0.05.

In this study, we aimed to specify DEGs in PC, compared to the normal tissue, regarding their plausible roles in the underlying pathways during cancer pathogenesis (Figure 1A). Considering the possible bias in PC incidence and survival rates between males and females, we have hypothesized that sex may affect these genes. Using microarray data and functional enrichment analysis, several genes and biological pathways were found to be possibly related to gender and basic mechanisms in PC.

### 2.1 Microarray datasets and analysis

The gene expression datasets from different microarray platforms related to PC were collected from the Gene Expression Omnibus (GEO) database (Table 1) (Pei et al., 2009; Barrett et al., 2013; Lunardi et al., 2014). Subsequently, GEO2R (http://www.ncbi.nlm.nih.gov/geo/geo2r/) was used to obtain DEGs in female and male patients relative to their matching controls, by using the linear models for microarray analysis (limma). As limma needs the data values to be in the log space, GEO2R automatically performs a log2 transformation on the selected sample values, which are specified not to be in log space (Smyth, 2004). The overall age range for females and males was 38–79 and 49–84, respectively. The absolute amount of LogFC value ≥ 1 or ≤ −1 was chosen as the cutoff criterion for the up- and downregulated genes, respectively, using a *p*-value of ≤ 0.05. The DEGs were then separately extracted in each gender relative to their corresponding controls.

**TABLE 1:** Specifications of selected studies based on microarray data.

Disease type	Source of sample	Series ID	Platform ID	Number of cases		Age range		Number of controls		Platform name	Year
				F	M	F	M	F	M		
PC	Pancreatic tumor	GSE16515	GPL570	14	22	57–79	49–84	4	12	[HG-U133_Plus_2] Affymetrix Human Genome U133 Plus 2.0 Array	2009
PDAC	Pancreatic tumor	GSE55643	GPL6480	17	28	UD	UD	4	4	Agilent-014850 Whole Human Genome Microarray 4 × 44K G4112F	2014
PC	Pancreatic Tumor	GSE22780	GPL570	5	3	38–73	59–69	5	3	[HG-U133_Plus_2] Affymetrix Human Genome U133 Plus 2.0 Array	2011

PDAC, pancreatic ductal adenocarcinoma; UD, undetermined.

### 2.2 Functional enrichment analysis

Applying Venn diagram version 2.1 (https://bioinfogp.cnb.csic.es/tools/venny/), similar up- and downregulated genes (U/DRGs) for each gender between all studies and the ones between both sexes were specified (Figure 1B) (Oliveros, 2007). The Database for Annotation, Visualization, and Integrated Discovery (DAVID) (Huang et al., 2009a; Huang et al., 2009b) and the Enrichr database (https://maayanlab.cloud/Enrichr/) (Chen et al., 2013; Kuleshov et al., 2016; Clarke et al., 2021) were used to determine KEGG (Kyoto Encyclopedia of Genes and Genomes) pathways (KEGG_2016_Human) for similar U/DRGs in both sexes (*p*-value ≤0.05). The KEGG database was used to interpret the data in terms of general schemes (Kanehisa et al., 2019).

To identify the genes with AREs or EREs, the list of restricted expressed genes to each gender specifically was submitted in the Enrichr database. The ChEA 2016 library was used to determine genes with the ability to bind to AR or ER, that is, possess AREs or EREs (*p*-value ≤ 0.05). In addition, these genes were investigated in terms of their location on any of the sex chromosomes, using the biological database network (bioDBnet) (https://biodbnet-abcc.ncifcrf.gov/db/db2db.php), to further assess the possibility of their affection in a gender-specific manner (Mudunuri et al., 2009). The Pancreatic Expression Database (PED) (https://www.pancreasexpression.org/) was used to investigate the possible correlation of expression profiles between upregulated ARE-containing genes in male patients with several top mutated genes in PC, in male and female patients from The Cancer Genome Atlas (TCGA) data source (Marzec et al., 2018). Moreover, PED was used to generate plots for assessing the relationship between these gene expressions and survival according to the TCGA data source (Marzec et al., 2018).

Finally, the TRANSFAC predicted transcription factor targets dataset (https://maayanlab.cloud/Harmonizome/dataset/TRANSFAC + Predicted + Transcription + Factor + Targets) was used to find potential transcription factors (TFs) among putative sex-affected gene candidates (Matys et al., 2003; Matys et al., 2006). The enriched Gene Ontology (GO) terms (biological process (BP), cellular component (CC), and molecular function (MF)) for ARE/ERE-containing genes were identifiedusing the Enrichr database (*p*-value ≤ 0.05).

### 2.3 Protein–protein interactions in sex-related U/DRGs

To establish protein–protein interactions (PPIs) and to subsequently identify the possible hubs in U/DRGs, for female and male patients, individually, the Search Tool for the Retrieval of Interacting Genes (STRING) database (https://string-db.org/) was used to analyze the data (score ≥0.4) (Szklarczyk et al., 2021). In addition, the HIPPIE dataset (http://cbdm-01.zdv.uni-mainz.de/∼mschaefer/hippie/) (Alanis-Lobato et al., 2016) and the BisoGenet tool (Martin et al., 2010) were also used to draw interactions between proteins. The identified PPIs from three data sources were then merged, and topological parameters, such as degree, and centrality measures, including betweenness centrality (BC), for undirected and directed networks, were computed, using a Cytoscape network analyzer software version 3.8.0. Proteins with at least a 10-degree score were specified as hub proteins. Also, the cutoff value ≥ 0.05 was used for BCs to specify bottleneck proteins, that is, network proteins that have many shortest paths going through them (Yu et al., 2007).

## 3 Results

### 3.1 DEG analysis in association with sex

Upon extraction of DEGs in three independent studies, considering both female and male patients’ data, (Supplementary Table S1–3), it was shown that 120 up- and 74 downregulated genes were common in male patients. The number of common U/DRGs between females was 49 and 3, respectively. A comparison between the two genders demonstrated that only *AHNAK2*, *C19orf33*, *KCNN4*, *MTMR11*, *NQO1*, *PHLDA2*, and *SYTL2* were common in the URGs, and no common genes were observed in the DRGs’ list (Table 2) (Figure 1B).

**TABLE 2:** List of common genes between all studies for each gender and both (genes which may be affected by gender are specified in bold, and GLI2 targets have been underlined).

Exp	Common	Just in male				Just in female	
**Upregulated**	*AHNAK2*	*ABCG1*	***EFNB2*** ^*ARE*^	*LOC100127972*	*RRAS*	*ABHD17C*	*MUC5AC*
	*C19orf33*	*ABLIM3*	*EHD1*	*LOX*	*RUNX1-IT1*	*ADGRF1*	*NMU*
	*KCNN4*	*ANO1*	***ELF4*** ^*X*^	*LPCAT4*	*S100A6*	*ARL14*	*PLEK2*
	*MTMR11*	*ANTXR1*	***EPHA4*** ^*ARE*^	*LRRC8A*	*S100P*	*ASPHD2*	*PSCA*
	*NQ O 1*	*ANTXR2*	***FBXO32*** ^*ARE*^	*LRRFIP1*	*SAMD9*	*CEACAM5*	*PTK6*
	*PHLDA2*	***ANXA1*** ^*ARE*^	***FN1*** ^*ARE*^	*LY6E*	*SCD*	*CTSE*	*RHBDL2*
	*SYTL2*	*AREG*	*FSCN1*	***MALL*** ^*ARE*^	*SERPINH1*	*DPCR1*	***SALL4*** ^*ERE*^
		*ARHGAP26*	*FXYD5*	*MDK*	***SH3KBP1*** ^*X*^	***ELOVL6*** ^*ERE*^	*SDR16C5*
		*ARRDC2*	*FZD2*	*MEGF6*	***SIM2*** ^*ARE*^	*EPS8L1*	*SERPINB5*
		***BICD1*** ^*ARE*^	*GBP1*	*MGLL*	***SLC24A3*** ^*ARE*^	*EZR*	*SGPP2*
		*BID*	*GJB2*	*MICALL2*	*SLC O 3A1*	*FA2H*	*SLC4A11*
		*BST2*	*GJB3*	*MMP11*	*SLFN13*	*GALNT5*	***SLC6A14*** ^*X*^
		***CALD1*** ^*ARE*^	***GLI2*** ^*ARE*^	*MX1*	*SPARC*	*ID1*	*SLC O 4A1*
		*CD109*	*GPRC5A*	*MYOF*	***SRPX2*** ^*X*^	*IL1RN*	*STYK1*
		*CD55*	*HK2*	***NDC80*** ^*ARE*^	*STX1A*	*KCNK1*	*SULT1C2*
		*CDH3*	*IFI44L*	***NHS*** ^*X*^	***SULF1*** ^*ARE*^	*LAMB3*	***TFF1*** ^*ERE*^
		*COL1A1*	*IGF2BP3*	*NOX4*	***SULF2*** ^*ARE*^	*LAMC2*	*TMPRSS3*
		***COL1A2*** ^*X*^	***IGFBP3*** ^*ARE*^	*NT5E*	***TACC3*** ^*ARE*^	*MCU*	*TRIM29*
		***COL3A1*** ^*ARE*^	*IGFBP5*	*NUAK1*	*THBS2*	*MIA*	*TRIM31*
		***COL5A1*** ^*ARE*^	*IL1R2*	***OSBPL10*** ^*ARE*^	*TNFAIP2*	*MMP28*	*TSPAN1*
		*COL5A2*	*INHBA*	***PALLD*** ^*ARE*^	*TNFAIP6*	*MST1R*	*VSIG2*
		*COR O 2A*	***ISG15*** ^*ARE*^	***PDLIM7*** ^*ARE*^	*TPM2*		
		*CRIP1*	*ISG20*	*PFKP*	*TPM4*		
		*CTHRC1*	*ITPR3*	*PLAU*	*UPP1*		
		*DDX60L*	*KIF26B*	*PMEPA1*	*VCAN*		
		*DKK3*	*KLK10*	*PPARG*	*WNT2*		
		*DNAJA4*	*KYNU*	*RAB31*	*XAF1*		
		*DPYSL3*	***LAMA3*** ^*ARE*^	*RAPH1*			
		***EDNRA*** ^*ARE*^	*LCK*	*RNF213*			
**Downregulated**	*—*	***ABAT*** ^*ARE*^	*ER O 1B*	*NR5A2*	***SLC16A10*** ^*ARE*^ */* ^*ERE*^	*BCL11A FGFR1 GNG7*	
		***ANPEP*** ^*ARE*^	*F11*	*NRCAM*	***SLC1A2*** ^*ARE*^		
		*AQP12B*	***FGL1*** ^*ERE*^	***NUCB2*** ^*ARE*^	***SLC25A15*** ^*ERE*^		
		***BHLHA15*** ^*ARE*^	*FKBP11*	***PAIP2B*** ^*ARE*^	*SLC25A45*		
		*BNIP3*	***GAS2*** ^*ARE*^	*PDCD4*	*SLC39A5*		
		*BTG2*	***GATM*** ^*ARE*^	*PDIA2*	***SLC39A8*** ^*ERE*^		
		*C5*	***GCAT*** ^*ERE*^	***PELI2*** ^*ARE*^ *PM20D1*	***SLC4A4*** ^*ARE*^		
		***CBS*** ^*ERE*^	*GLS2*	***PNLIPRP1*** ^*ARE*^	*SLC7A2*		
		***CHRM3*** ^*ARE*^	***GMNN*** ^*ARE*^	*PPP2R2D*	***STXBP6*** ^*ARE*^		
		*COCH*	***GNMT*** ^*ERE*^	***PRDX4*** ^*X*^	*SYBU*		
		*CTH*	*GSTA3*	***PRLR*** ^*ARE*^	*TCEA3*		
		***CTTNBP2*** ^*ARE*^	*GUCA1C*	*PROX1*	*TDH*		
		*CYP4V2*	*JADE1*	*RAB26*	***TEX11*** ^*X*^		
		***DMD*** ^*X*^	*KLK1*	*SEC11C*	*TMED6*		
		***DNASE1*** ^*ARE*^	***LPAR3*** ^*ARE*^	***SEL1L*** ^*ARE*^	***TMEM56*** ^*ARE*^		
		*DPP10*	*MPP7*	***SEMA6D*** ^*ARE*^	*TPCN2*		
		*ENPP1*	*MPV17L*	*SERPINI2*	*TPST2*		
		*ENTPD3*	*MUC15*		*TRIM50*		
		***EPB41L4B*** ^*ARE*^	***NEDD4L*** ^*ARE*^				

ARE, androgen response element; ERE, estrogen response element; X, X chromosome.

### 3.2 Discovering the gender-related DEGs

Following the enrichment analysis on male-related URGs, it was specified that *ANXA1*, *BICD1*, *CALD1*, *COL3A1*, *COL5A1*, *EDNRA*, *EFNB2*, *EPHA4*, *FBXO32*, *FN1*, *GLI2*, *IGFBP3*, *ISG15*, *LAMA3*, *MALL*, *NDC80*, *OSBPL10*, *PALLD*, *PDLIM7*, *SIM2*, *SLC24A3*, *SULF1*, *SULF2*, and *TACC3* have ARE, whereas no genes with ERE were found in this class. Moreover, *COL1A2*, *ELF4*, *SH3KBP1*, *SRPX2*, and *NHS* were shown to be on the X chromosome. Regarding female-related URGs, *ELOVL6*, *SALL4*, and *TFF1* have ERE, whereas *SLC6A14* is located on the X chromosome. Among the male-related DRGs, *ABAT*, *ANPEP*, *BHLHA15*, *CHRM3*, *CTTNBP2*, *DNASE1*, *EPB41L4B*, *GAS2*, *GATM*, *GMNN*, *LPAR3*, *NEDD4L*, *NUCB2*, *PAIP2B*, *PELI2*, *PNLIPRP1*, *PRLR*, *SEL1L*, *SEMA6D*, *SLC1A2*, *SLC4A4*, *STXBP6*, and *TMEM56* were found to have ARE, whereas *CBS*, *FGL1*, *GCAT*, *GNMT*, *SLC25A15*, and *SLC39A8* contained ERE. *SLC16A10* has both ARE and ERE. Furthermore, *DMD*, *PRDX4*, and *Tex11* are located on the X chromosome*.* There were no female-related DRGs having ARE or ERE. In addition, no genes were specified to be located on the X chromosome (Table 2).

**FIGURE 2:**
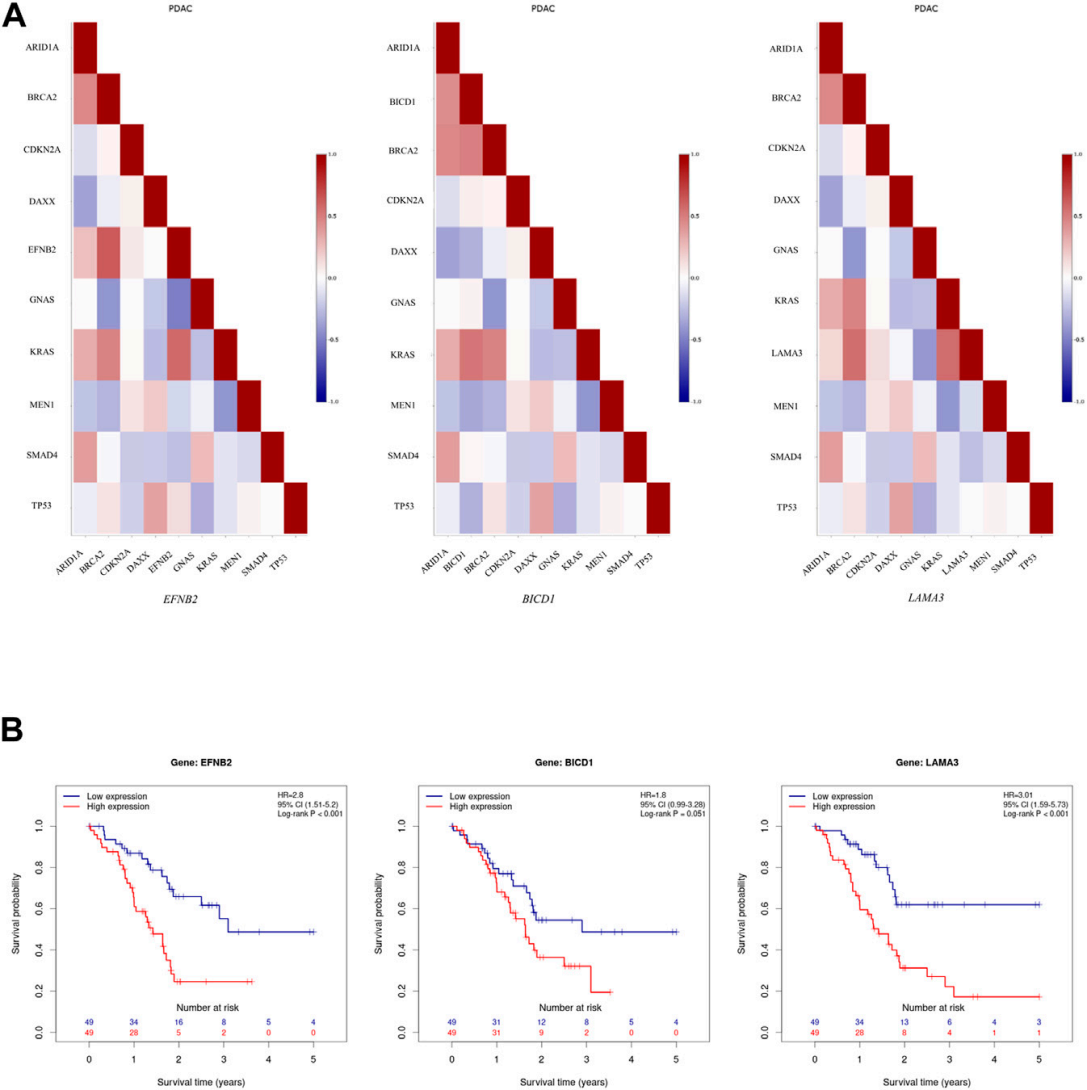
Further assessment of upregulated ARE-containing genes in male patients among the male PC patients from TCGA data source. **(A)** Evaluating the correlation of upregulated ARE-containing genes with top mutated genes in PC showed that *EFNB2*, *BICD1*, and *LAMA3*, have a high positive correlation with *KRAS* expression among male PC patients from TCGA data source. The color of each cell indicates correlation coefficient between corresponding genes labelled on the *x*-axis and *y*-axis. **(B)** The survival analysis on*EFNB2*, *BICD1*, and *LAMA3* in male PC patients from TCGA data source showed a decrease in survival probability upon their high expression. Data is obtained from Pancreatic Expression Database (PED) (https://www.pancreasexpression.org/).

It seems that ARE-containing genes that were upregulated in male patients have a higher correlation in their expression profiles with each other, in comparison with female PC patients from the TCGA data source, based on the Pearson Product Moment Correlation Coefficient (PMCC) value (Supplementary Figure S1). Also, assessing the correlation between these gene expressions and several top mutated genes in PC confirmed their high correlation with *KRAS*, among others (Supplementary Figure S2A). Of note, *EFNB2*, *BICD1*, and *LAMA3* showed a high positive correlation with *KRAS* (Figure 2A). Meanwhile, investigating downregulated genes in male patients showed a negative correlation with *KRAS* (Supplementary Figure S2B). Furthermore, the survival analysis showed association between lower survival probability and upregulation of several of these genes in male PC patients according to the TCGA data source (Supplementary Figure S3). Interestingly, the decreased survival probability was also clearly showed for *EFNB2*, *BICD1*, and *LAMA3* (Figure 2B).

#### 3.2.1 Sex-possibly affected genes can act as transcription factors

By annotating a gene function using Entrez Gene Summary and PIR Summary in DAVID for genes with sex hormone regulating elements, it was specified that *GLI2* ^ARE^, *SIM2* ^ARE^, and *ELF4* ^X^ in male-related URGs, *SALL4* ^ERE^ in female-related URGs, and *BHLHA15* ^ARE^ in the male-related downregulated class have the transcription regulatory function. Some of GLI2 targets exist within the list of male URGs, including *ABCG1*, *ARRDC2*, *C19orf33*, *COL5A1* ^ARE^, *FZD2*, *GJB2*, *ITPR3*, *LY6E*, *MMP11*, *NDC80* ^ARE^, *NHS* ^X^, *PALLD* ^ARE^, and *WNT2*. In addition, based on the TCGA data source, it seems that *GLI2* expression is slightly higher in male PC patients in comparison with the female patients (Supplementary Figure S4).

#### 3.2.2 Enrichment analysis of gender-related DEGs in male and female patients

**FIGURE 3:**
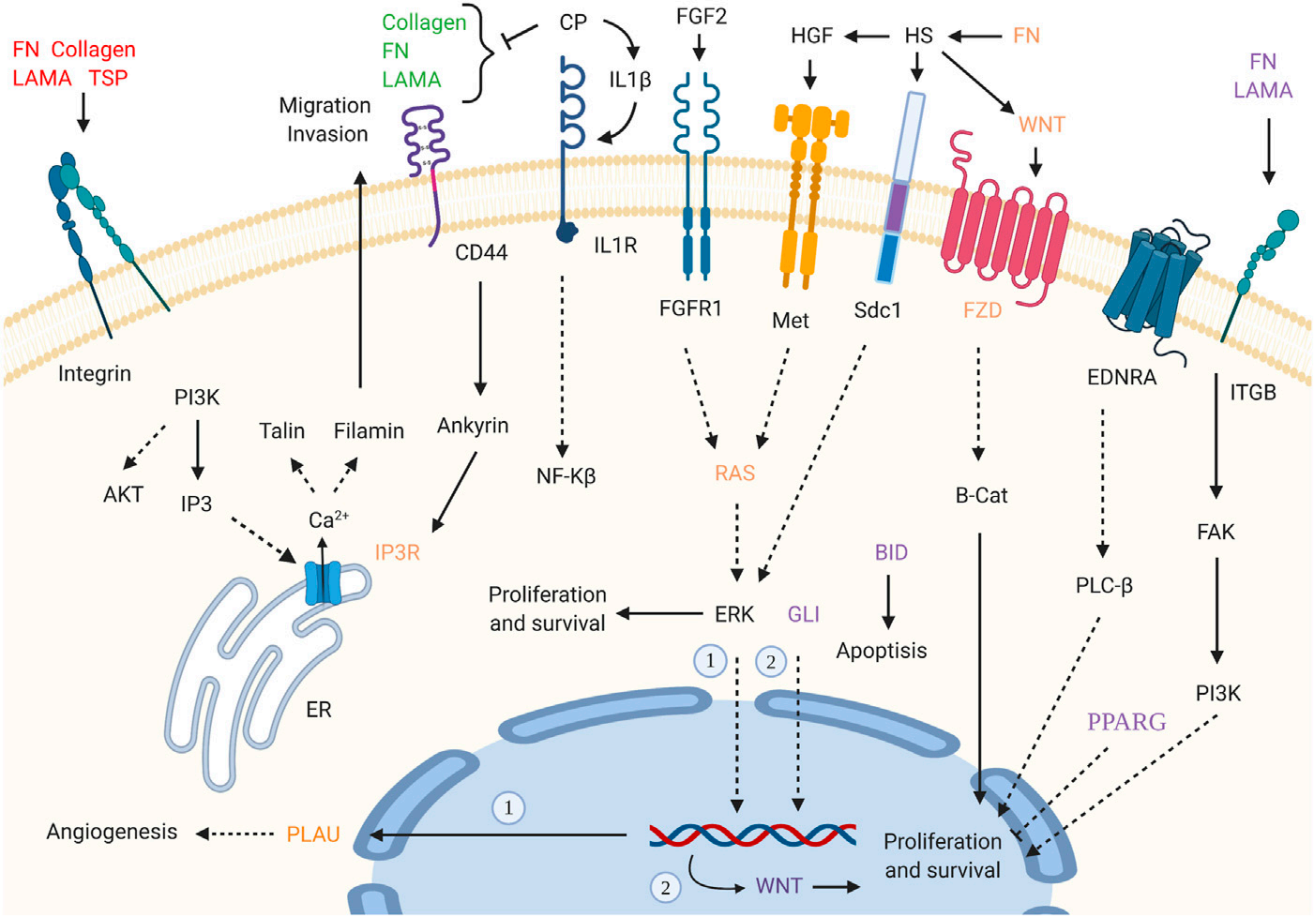
Enriched signaling pathways for URGs in male pancreatic cancer (PC) patients. Interaction of extracellular matrix (ECM) components and proteoglycans with intracellular signaling pathways, such as WNT signaling, and cell proliferation, ERK signaling, and angiogenesis has been implicated. GLI2 is also highlighted in pathways in cancer_hsa05200. (*p*-value ≤0.05). “Created with BioRender.com.”

**FIGURE 4:**
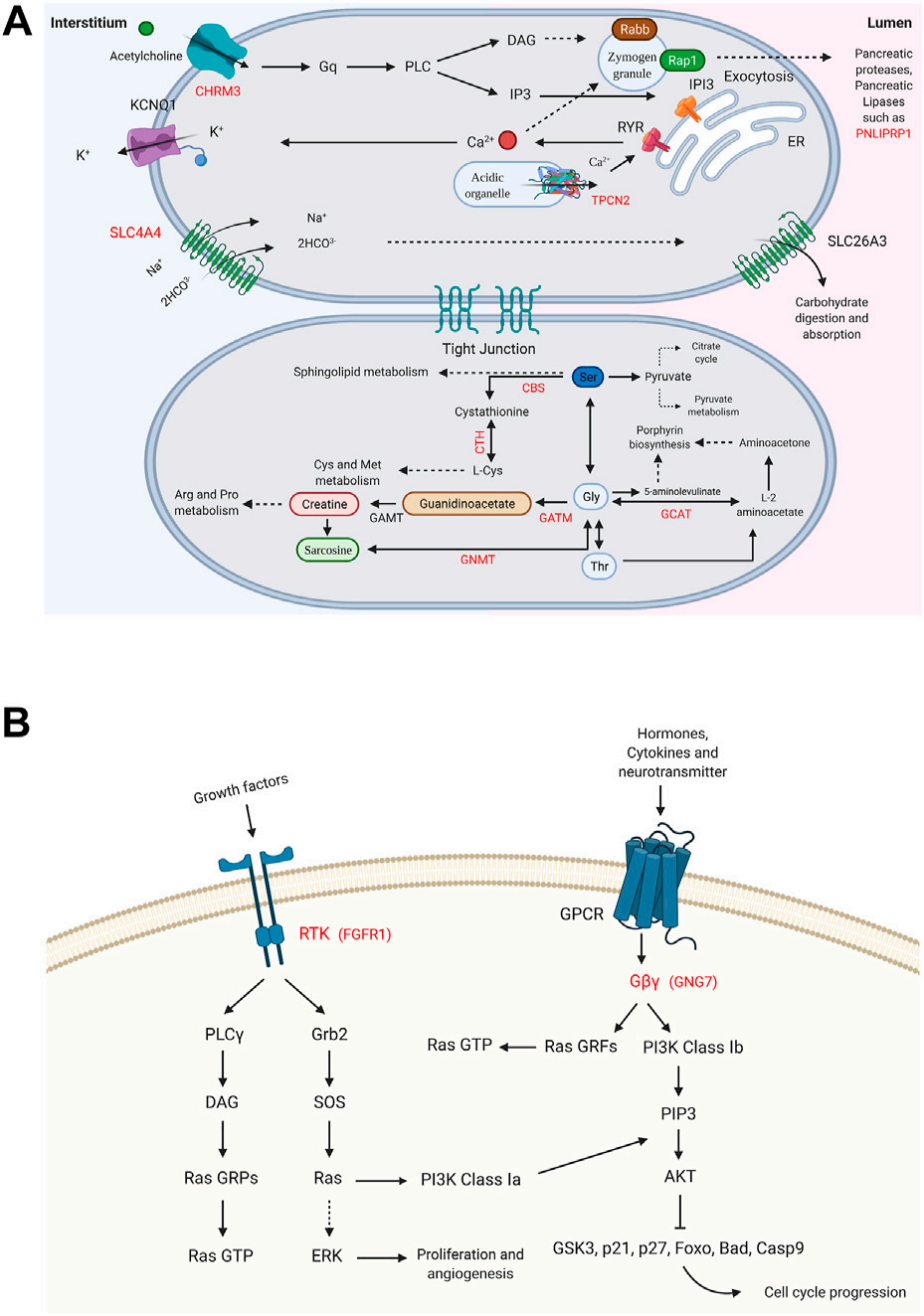
Enriched signaling pathways for the DRGs in male and female pancreatic cancer (PC) patients. **(A)** Glycine, serine, and threonine metabolism_hsa00260 and pancreatic secretion_hsa04972 pathways are shown for DRGs in male PC patients. **(B)** Ras signaling pathway_hsa04014 and PI3K-Akt signaling pathway_hsa04151 are specified for FGFR1 and GNG7, the DRGs in female PC patients. (*p*-value ≤0.05). “Created with BioRender.com.”

**FIGURE 5:**
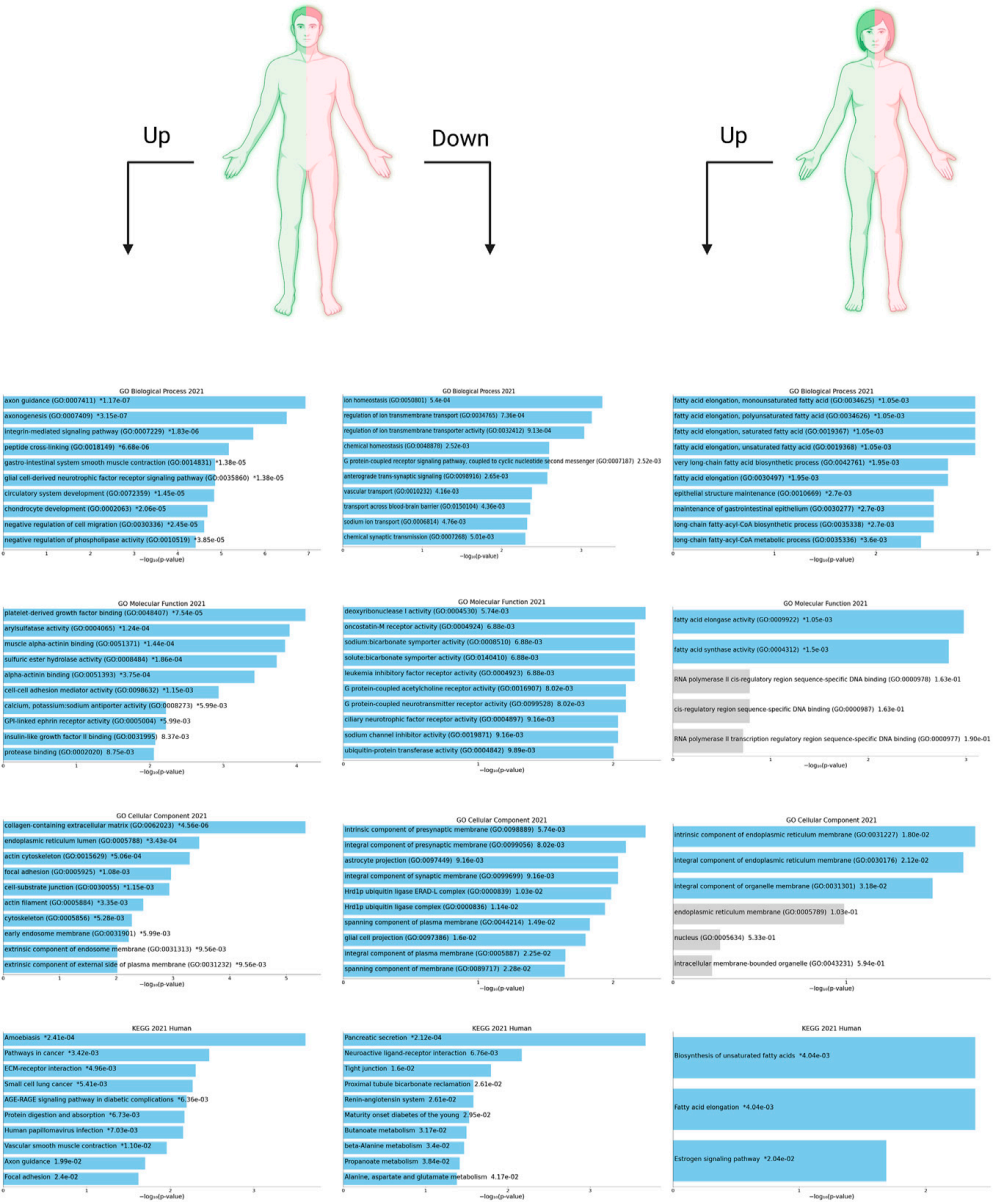
Top 10 enriched Gene Ontology (GO) terms (i.e., biological process (BP), molecular function (MF), and cellular component (CC)) and KEGG pathways have been shown for ARE-containing up- and downregulated genes (U/DRGs) in male PC patients. Also, the top enriched GO terms and KEGG pathways are listed for ERE-containing URGs in female PC patients. The corresponding *p*-values are shown. Blue bars correspond to terms with significant *p*-values (<0.05). An asterisk (*) next to a *p*-value indicates the term also has a significant adjusted *p*-value (<0.05). All data shown here are retrieved from the Enrichr database (https://maayanlab.cloud/Enrichr/) and visualized using Enrichr Appyter.

Supposing that gender-related U/DRGs in PC patients may reveal new insights toward the possible association of sex and PC progression or, cues regarding its development, the respected signaling pathways for each group have been investigated, based on the retrieved data from DAVID and KEGG databases (Figures 3, 4). Furthermore, according to specified GO terms for ARE-containing up- and downregulated genes in male patients and ERE-containing upregulated genes in female patients (Figure 5), it was shown that ARE-containing URGs in males are significantly involved in axon guidance (GO:0007411) (*p*-value ≤ 0.01) (Supplementary Table S4).

Pathways with a possible role in disease development were deduced for male-related U/DRGs and female-related DRGs (*p*-value ≤ 0.05) (Supplementary Table S5). No pathways with significant relation to female-related URGs were observed. Among the URGs in male patients, extracellular matrix (ECM) components (COL3A1, COL1A2, COL1A1, COL5A2, COL5A1, LAMA3, FN1, and THBS2) and their deduced interaction with intracellular signaling pathways have been highlighted. The other enriched pathway, proteoglycans in cancer (proteoglycans in cancer_hsa05205), has also been specified with its implication in WNT signaling, cell proliferation, ERK signaling, and angiogenesis. Another indicated protein, GLI2, is also of note in pathways in cancer_hsa05200. It can mediate cell proliferation through *WNT* expression (Figure 3). The enriched pathways related to DRGs in male patients include glycine, serine, and threonine metabolism_hsa00260 and pancreatic secretion_hsa04972 (Figure 4A). Regarding the latter, it can be seen that through downregulation of CHRM3 and TPCN2 proteins, the secretion of pancreatic proteases and lipases would be affected, whereas the reduction in SLC4A4 protein can modify carbohydrate digestion and absorption. Finally, it is of note that downregulated proteins, FGFR1 and GNG7, in female patients are significantly related to the Ras signaling pathway_hsa04014 and the PI3K-Akt signaling pathway_hsa04151 (Figure 4B).

### 3.3 Hub and bottleneck proteins in U/DRGs

Considering the defined cutoffs, hub and bottleneck proteins were only detected for URGs in male patients (Supplementary Table S6). In addition, several proteins have been specified as both hubs and bottlenecks. These include FN1, CALD1, ISG15, SH3KBP1, COL1A1, PPARG, LOX, LCK, and SPARC.

## 4 Discussion

The prevalence of pancreatic adenocarcinoma is relativelyhigher in males compared to females in regions including theUnited Kingdom and the United States, where the samples used in this study were obtained from (McGuigan et al. 2018; Pourshams et al. 2019). In this regard, investigating the pathways that certain up- or downregulated proteins re involved in, for example, in male PC patients, may provide valuable information regarding the relation between sex and cancerspecific signaling pathways.

In our study, ECM–cell interactions and other closely linked pathways, such as focal adhesion, proteoglycans in cancer, and their subsequent signaling cascades, have been highlighted, respecting the URGs in male PC patients. However, it should be noted that, generally, one of the main hallmarks of the immunosuppressive microenvironment of PC in both sexes is the excessive deposition of ECM components (Orth et al., 2019). Among several components of ECM, collagens are the most abundant (Weniger et al., 2018). In our study five out of eight upregulated proteins involved in ECM–receptor interactions are collagens located in the interstitial space (COL1A2, COL1A1, COL3A1, COL5A2, and COL5A1) (Supplementary Figure S4). Interstitial collagens and mainly collagen type 1 have tumor-promoting properties, which exert proliferation and migration, among others (Weniger et al., 2018; Xu et al., 2019). To the best of our knowledge, no study has been conducted to investigate the interaction of AR and ECM in PC. Nevertheless, it has already been shown that the androgen and estrogen ratio can mediate the expression of COL3A1 in the rat brain, while its expression in females decreased (Yonehara et al., 2003). In addition, the diameter of collagen fibrils can be regulated through sex hormone levels in favor of males (Markova et al., 2004). However, DRGs in male patients were enriched in amino acid metabolism as well as protease and lipase secretion–related pathways. Specifically, downregulation of cystathionine-beta-synthase and cystathionine gamma-lyase in glycine, serine and, threonine metabolism pathways would most probably block L-cysteine production from serine and, therefore, affect cysteine and methionine metabolism negatively. Also, one of the interesting inferences is the possible reduction of creatine following the downregulation of glycine amidinotransferase (GATM), also known as L-arginine:glycine amidinotransferase, which is the rate-limiting step of creatine synthesis from arginine and glycine (Figure 4A). In general, we propose that downregulation of specific components in the mentioned pathways may grant certain advantages for PC progression in males, as their reduced levels have been confirmed in three independent studies and specifically among males.

Next, the association between the presence of ERE/ARE in the regulatory regions of U/DRGs and PC underlying mechanism was investigated in a gender-specific manner. Interestingly, we noticed that the biological process *axon guidance* has been significantly highlighted among the ARE-containing URGs, that is, *EFNB2*, *EPHA4*, *PALLD*, *LAMA3*, *PDLIM7*, and *GLI2*, among the male patients. Frequent and diverse somatic aberrations in genes involved in regulating axon guidance have already been demonstrated to be important in pancreatic carcinogenesis (Biankin et al., 2012). This, along with the observation that the number and size of intrapancreatic nerves increase in PDAC, a process called pancreatic cancer–associated neural remodeling (PANR) (Wakiya et al., 2021), underlines the possible importance of sex’s role in PC progression and how male hormones may affect the expression of involved genes. On the other hand, the presence of several TFs among the AER/ERE-containing genes may emphasize a more possible effect of sex on disease progression. For example, *GLI2*, among resulted ARE-containing URGs in males (AR_21915096_ChIP-Seq_LNCaP-1F5_Human), is a TF in the Hedgehog pathway and has long been shown to be associated with prostate cancer tumorigenesis, among others (Xia et al., 2020). Thus far, several studies have investigated GLI’s interaction with AR and demonstrated the resulted induction in the expression of GLI-dependent genes due to androgens (Li et al., 2018a). Although it has been shown that AR might also modulate transcription from *GLI2* (Li et al., 2014a), the effect of AR on the expression level of GLI proteins through ARE still needs to be further investigated. Also, several GLI2 targets, including *ABCG1*, *ARRDC2*, *C19orf33*, *COL5A1*, *FZD2*, *GJB2*, *ITPR3*, *LY6E*, *MMP11*, *NDC80*, *NHS*, *PALLD*, and *WNT2*, were shown to be upregulated in male patients. In general, targeting GLI2 seems a promising strategy that may result in the downregulation of a range of genes involved in various biological processes. The other resulted ARE-containing URG, *Single-minded 2 (SIM2)*, encodes a TF with a shown tumorgenesis activity in PC (DeYoung et al., 2003).

We also further investigated if the URGs on the X chromosome in male patients may have any possible association with a gender-related mechanism regarding cancer progression. While *SH3* domain-containing kinase binding protein 1 (SH3KBP1), Sushi Repeat Containing Protein X-Linked 2 (SRPX2), and NHS actin remodeling regulator (NHS) have been shown to have a variable escape from X chromosome inactivation (XCI), one copy of *E74 Like ETS Transcription Factor 4* (*ELF4*) is indeed inactive in females, due to XCI (Carrel and Willard, 2005; Tukiainen et al., 2017) and interestingly, as a TF, it can regulate its targets in a dose-dependent manner (Du et al., 2021). High ELF4 expression is shown to be related to worse disease outcomes in several cancers, including PC, although observations exist in accordance with its both oncogenic and tumor suppressor roles in different cancer types (Suico et al., 2017; Kafita et al., 2021). Still, there is much to be identified regarding the involved mechanism through which ELF4 regulates its downstream target; however, it gained our attention that this TF has been identified as one allelic-specific expression site (Chuang et al., 2017). The ASE bias toward the paternal (P) or maternal (M) alleles was described as one P and 2 M among three studies, that is, the Geuvadis RNA Sequencing Project at http://www.geuvadis.org/web/geuvadis/rnaseq-project, Li et al., 2014b’s study, and Cenik et al., 2015’s study. This can be interesting as males always receive the maternal X chromosome.

Finally, one important consideration is the possible effect of sex on the identified hub proteins such as those related to ECM, that is, fibronectin 1 (FN1; with possible ARE), collagen type I Alpha 1 (COL1A1), lysyl oxidase (LOX), interferon-stimulated gene 15 (ISG15; with possible ARE), and caldesmon gene1 (CALD1; with possible ARE) (Table 2)*.* Multiple lines of evidence have thus far demonstrated the effect of ECM dysregulation on tumor progression (Weniger et al., 2018). However, ECM seems to be affected by sex in different cell types, and the possible association between the androgen receptor pathway and collagen content has been demonstrated (Markova et al., 2004). In accordance, the physiological testosterone is also shown to be involved in enhancing ECM synthesis in certain cells (Bertolo et al., 2014). Also, *CALD1*, is an actin-linked regulatory protein with multiple functions in cell motility, such as migration, invasion, and proliferation (Mayanagi and Sobue, 2011). While this hub-bottleneck protein may have the potential to be among the candidate cancer drivers in PC, it has already been shown to be androgen sensitive (Erho et al., 2012; Dressler et al., 2021).

## 5 Conclusion

Given the relatively biased rates of incident and survival among male and female PC patients, in this study, we have tried to shed light on the possible relation between DEGs in PC and gender. The possible regulation of highly or lowly expressed genes in male or female pancreatic tumors through sex hormones can be of great value in finding the important molecular mechanisms which may make one gender more potent for developing this cancer. It can also help identifying potential gender-specific biomarkers for linking gender and the pancreatic carcinogenesis. In addition, considering the promising results of specific androgen receptor blockers such as flutamide in increasing the survival of PC patients in two clinical trials, the current study may have benefits in elucidating the potential mechanisms mediating this effect of androgen receptor blockers (Greenway, 2000). This may also show the possible advantage of certain therapies, such as hormone withdrawal therapy in PC patients, although the reports are conflicting in this regards. One interesting suggestion would be to check and screen these potential candidates and assess their expression profile and their downstream targets (in case of TFs) in male deriving tumor cells upon treating with androgen blockers while investigating the cancerous phenotype. Finally, it is of note to take a specific look at the TFs as the plausible targets of ERs and ARs and the regulators of a broad range of genes. Using high-throughput single cell RNA sequencing methods and identifying the master transcription factors with possible ARE/ERE through methods such as SCENIC would pave the way of finding other potential candidates in this regards. However, one main drawback of this study was the unavailability of the patients’ clinical characteristics in accordance with gender, which, therefore, limits the relevancy of the identified genes and the clinical outcomes. While this should to be considered in future studies, discovering key DEGs with relation to gender may help to develop new modeling platforms, detection approaches, and targeted therapies.

## Data Availability

The datasets presented in this study can be found in online repositories. The names of the repository/repositories and accession number(s) can be found in the article/Supplementary Material.
