# Supplementary material for: Gender-related differentially expressed genes in pancreatic cancer: possible culprits or accomplices?
Source: Front Genet. 2022 Oct 26;13:966941. doi: 10.3389/fgene.2022.966941 (PMC9643577; doi:10.3389/fgene.2022.966941)
Supplement: Supplementary file 3 [file DataSheet4.PDF]

GLI2

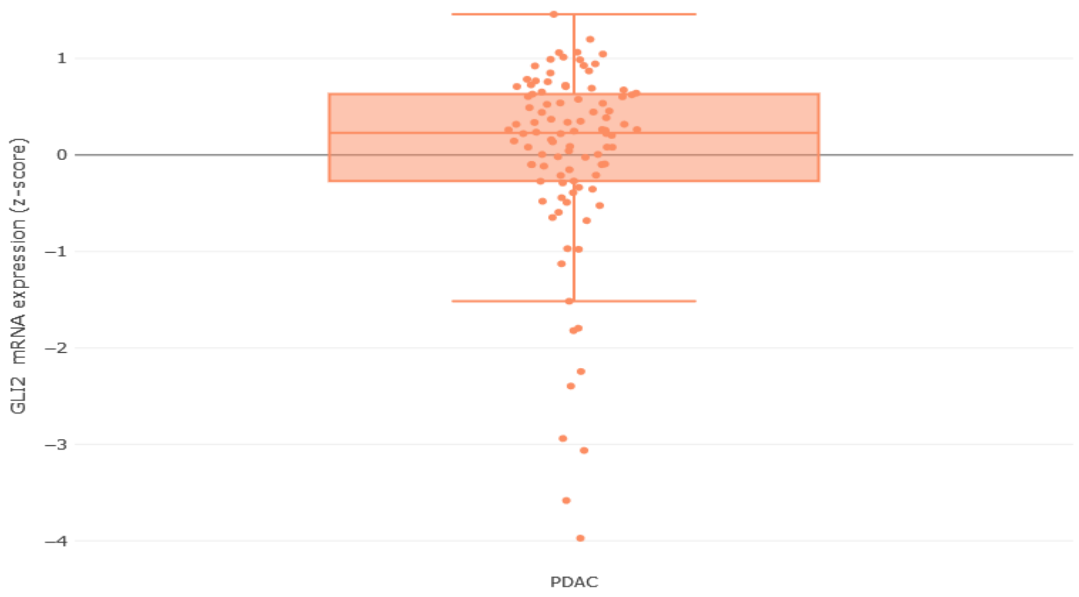

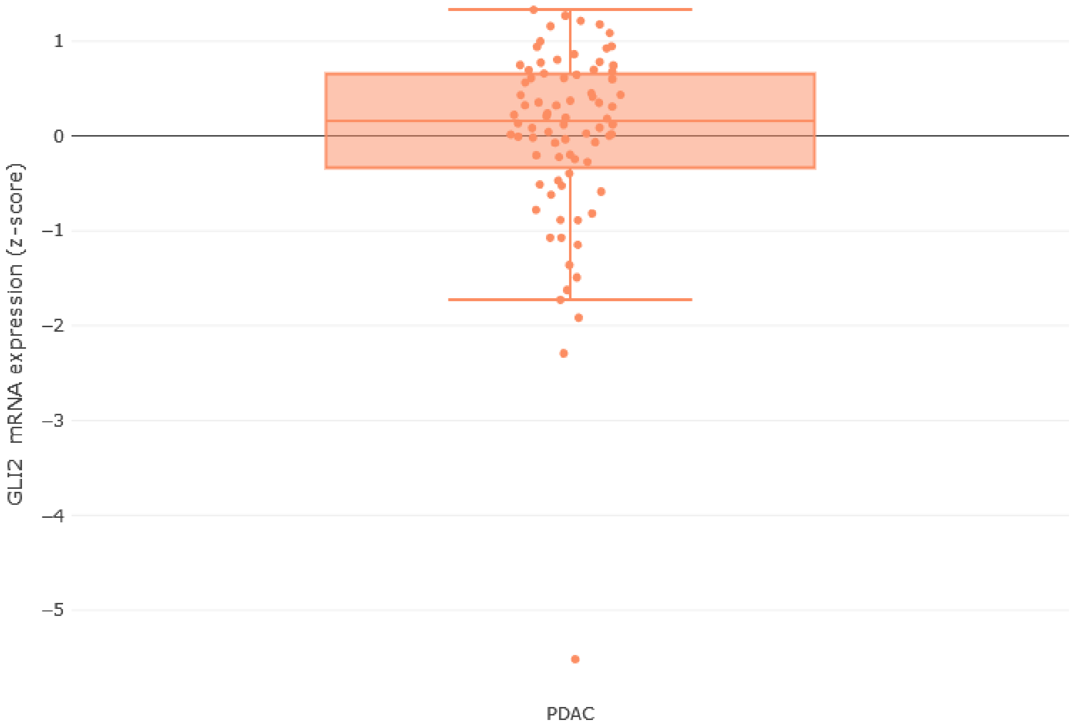

Male

Female

COL1A1

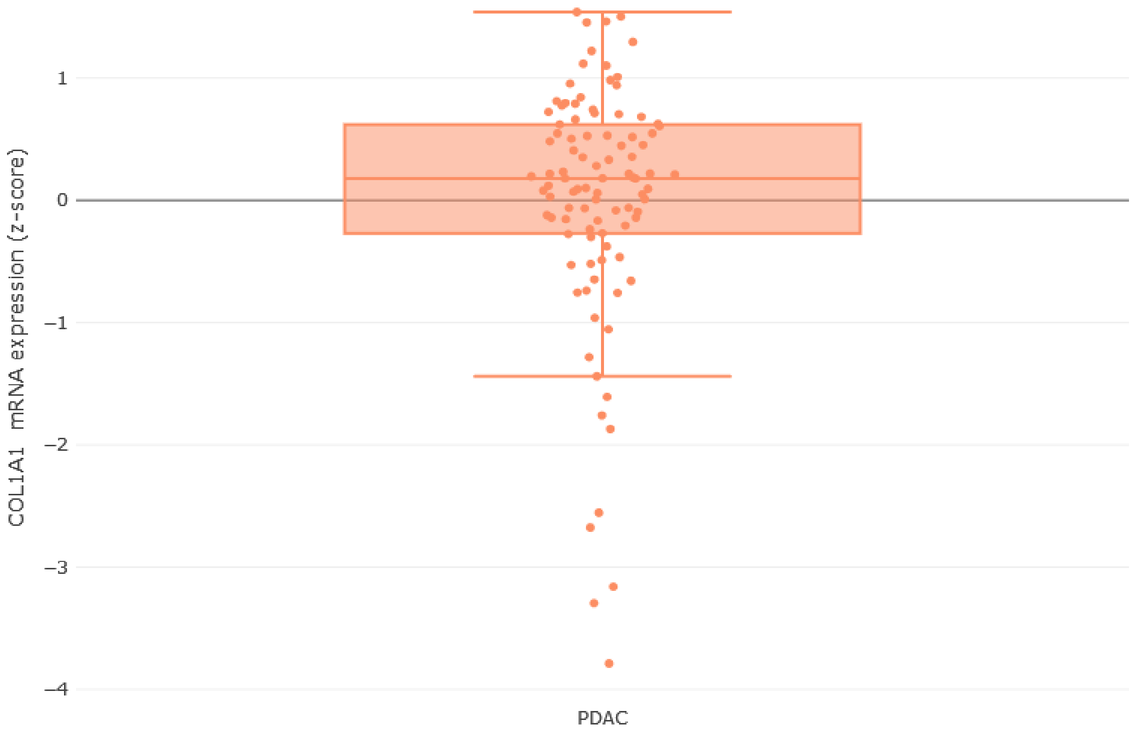

Male

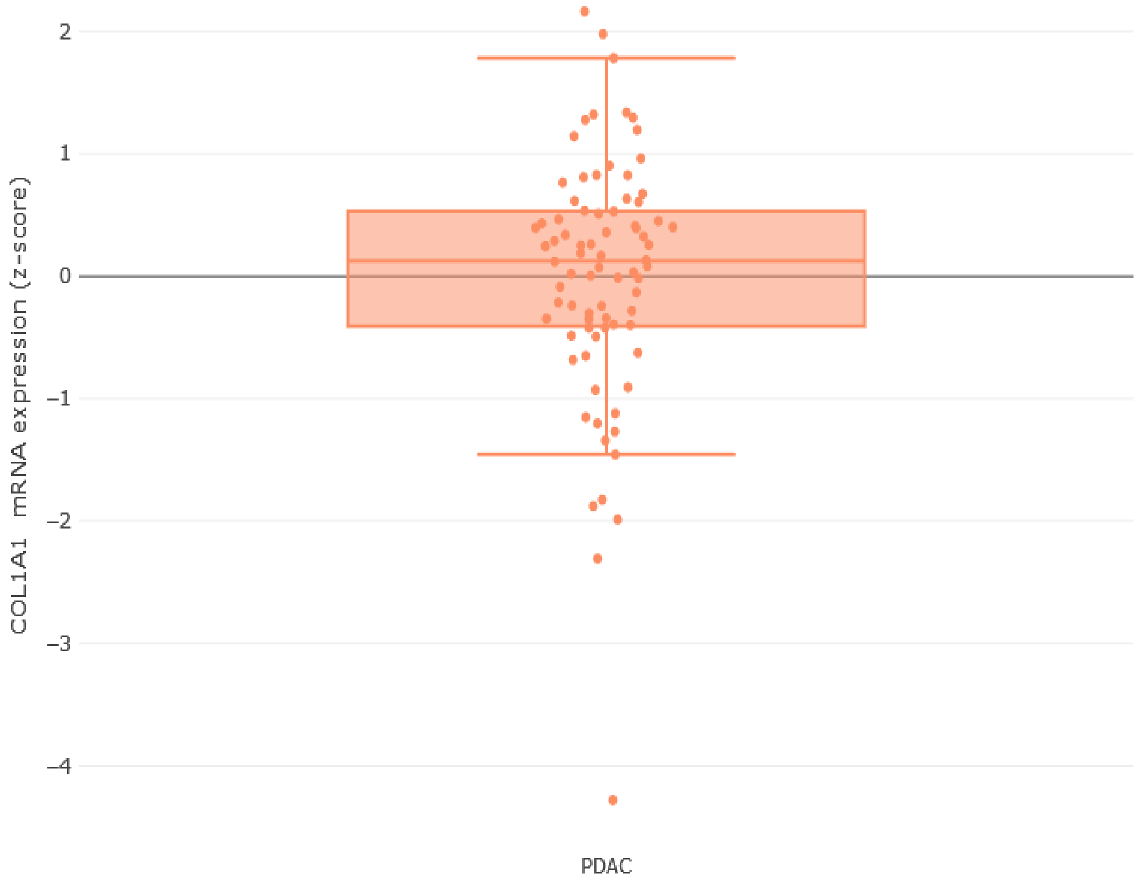

Female

COL5A1

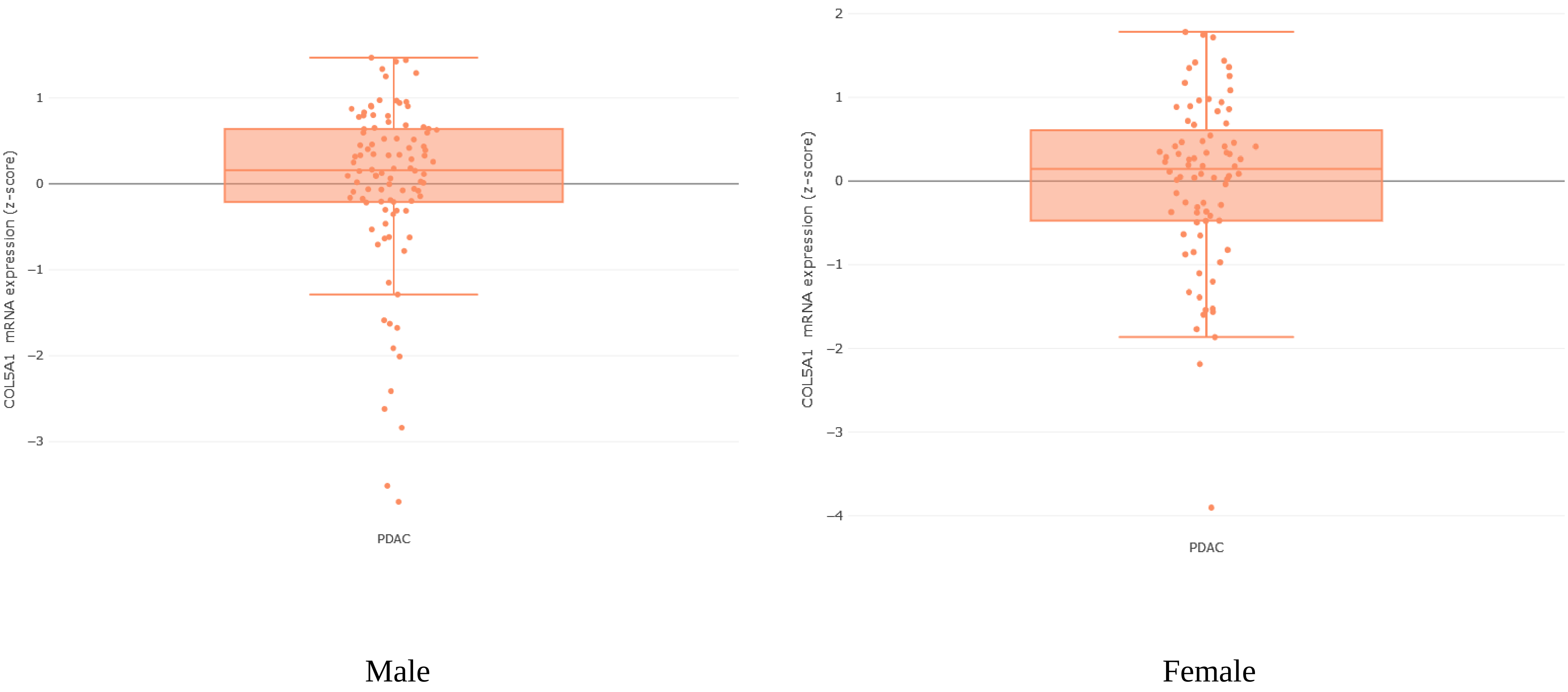

Supplementary Figure 4. Comparison of GLI2, COL1A1, and COL5A1 in male and female PC patients based on TCGA data source. The data is obtained from Pancreatic Expression Database (PED) (https://www.pancreasexpression.org/).
