# Supplementary material for: Gender-related differentially expressed genes in pancreatic cancer: possible culprits or accomplices?
Source: Front Genet. 2022 Oct 26;13:966941. doi: 10.3389/fgene.2022.966941 (PMC9643577; doi:10.3389/fgene.2022.966941)
Supplement: Supplementary file 6 [file Image2.pdf]

a.

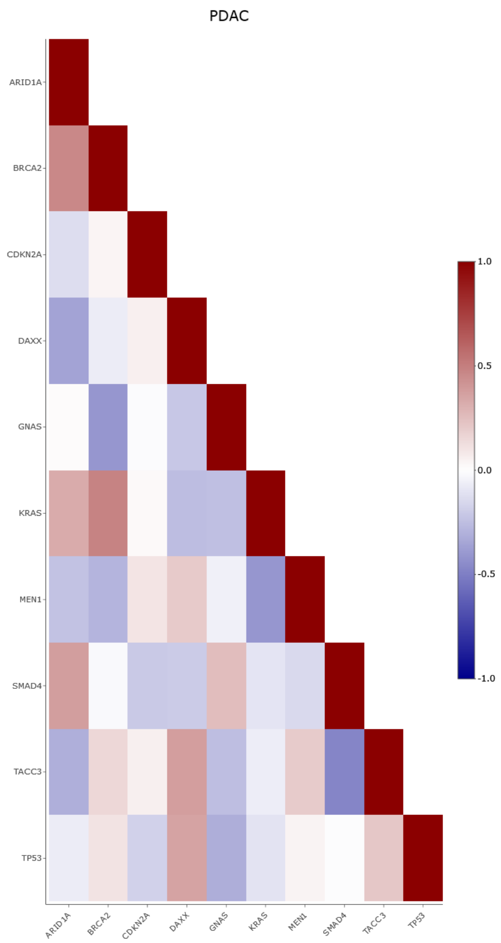

TACC3

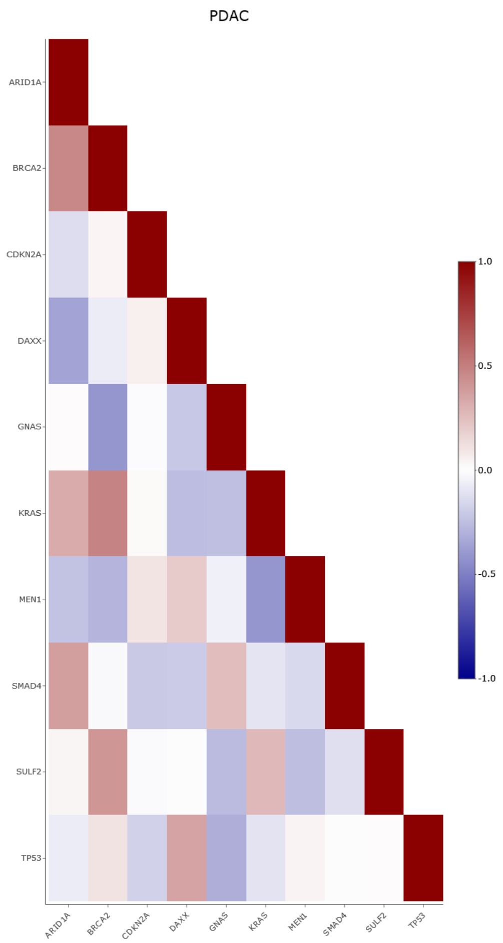

SULF2

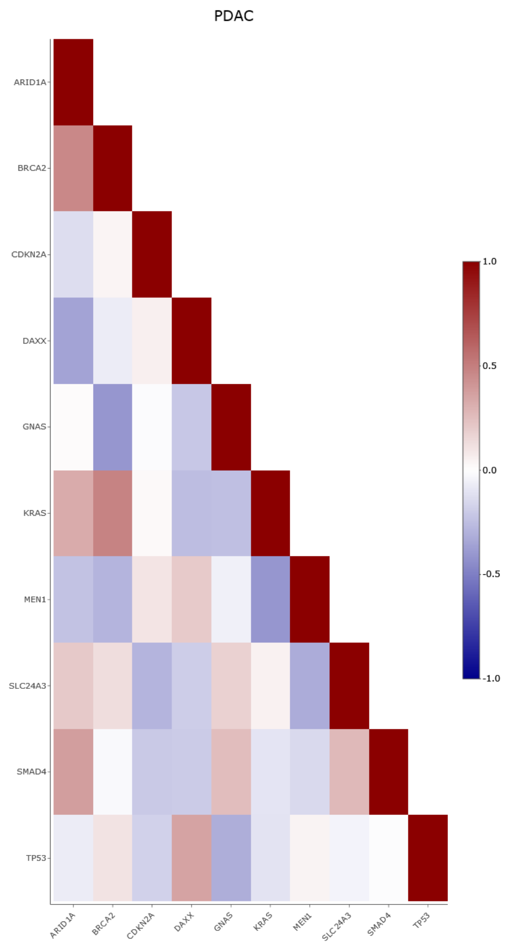

SLC24A3

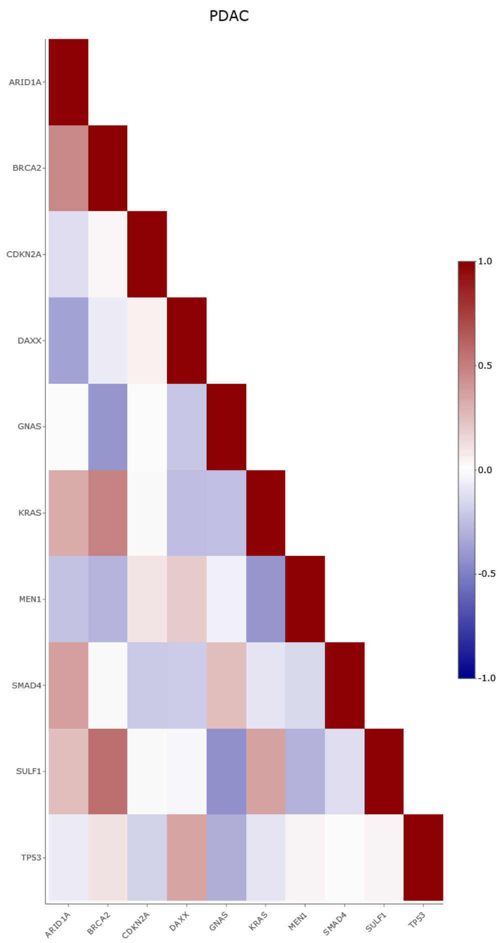

SULF1

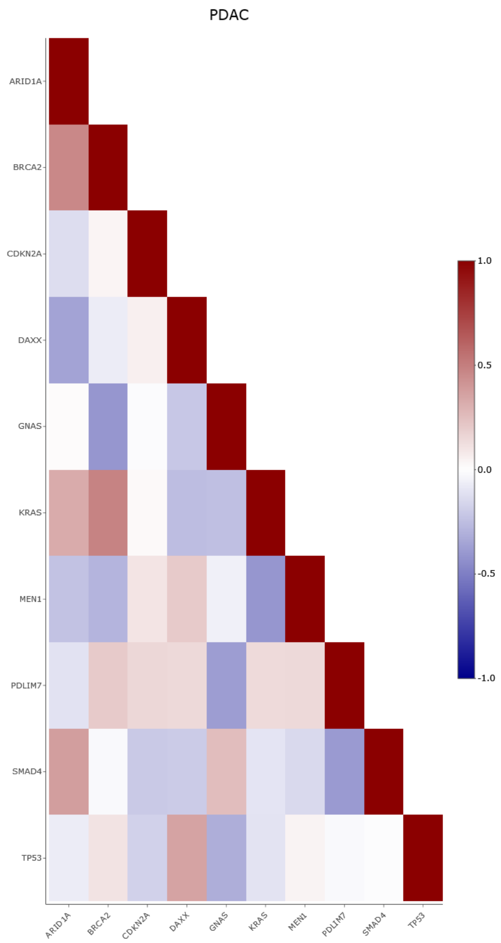

PDLIM7

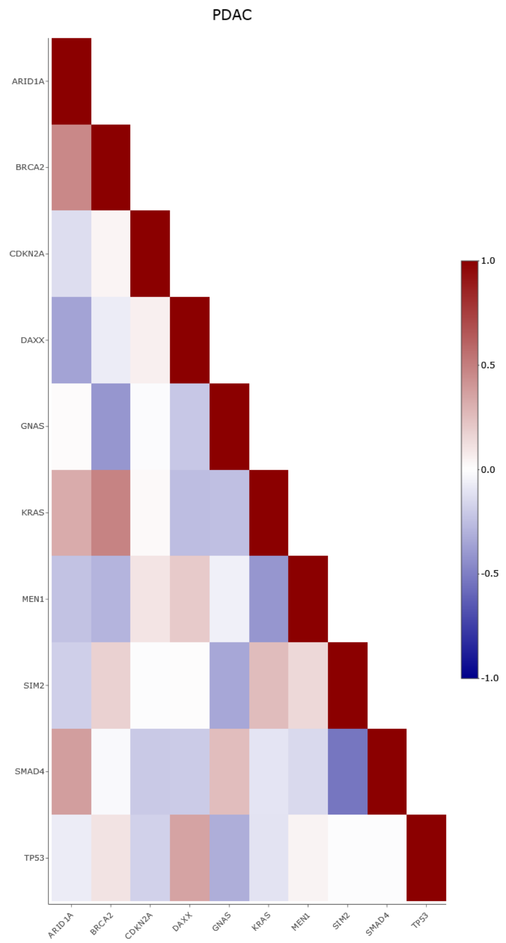

SIM2

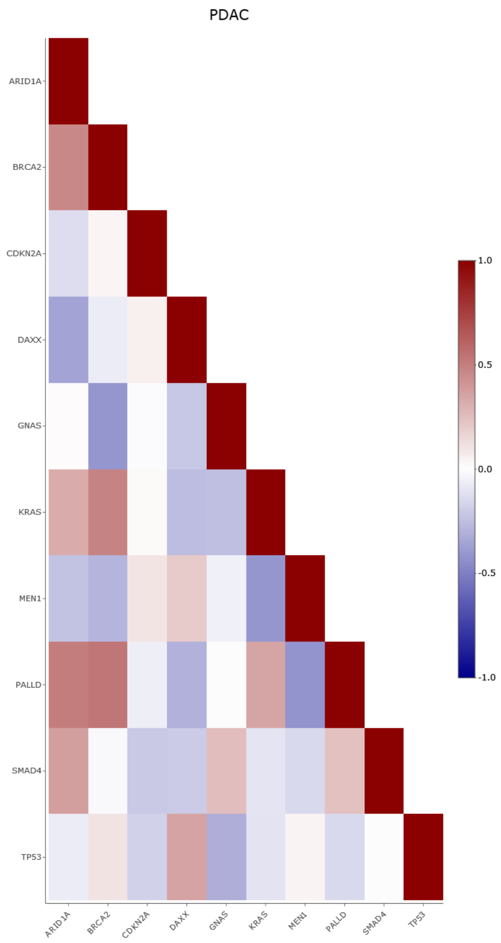

PALLD

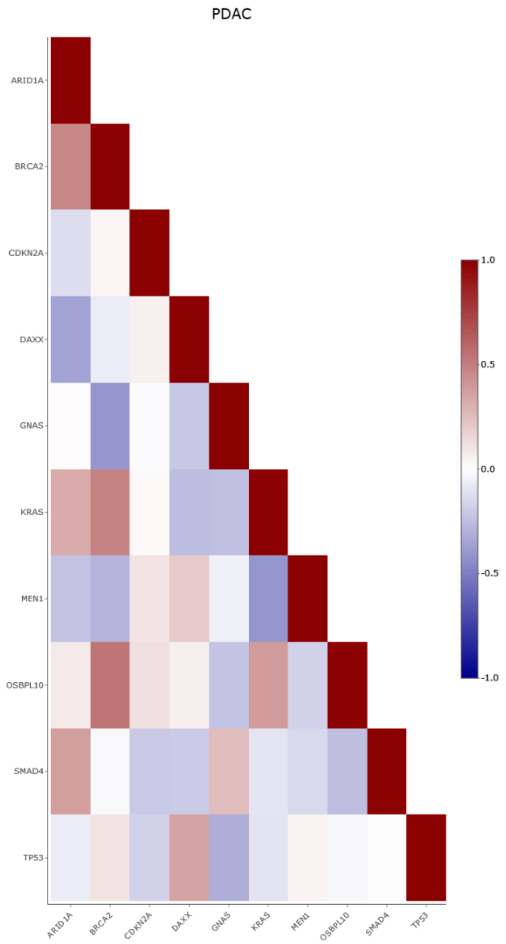

OSBPL10

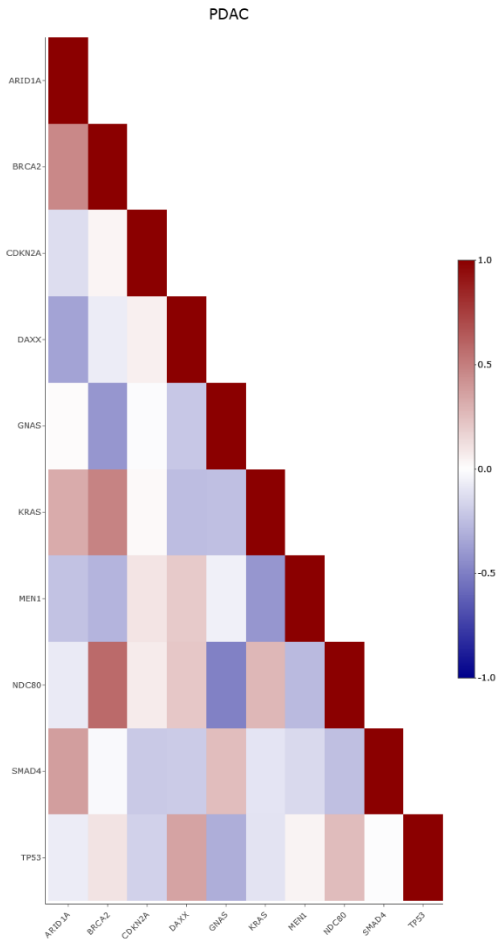

NDC80

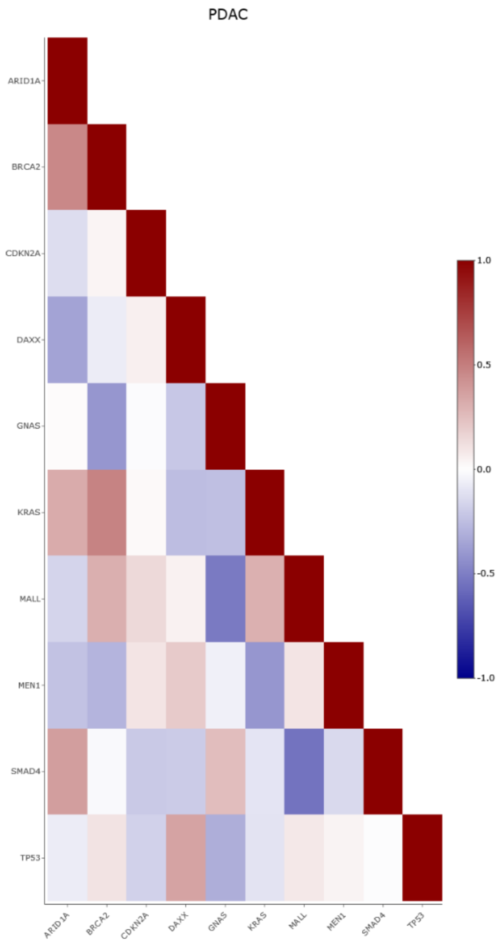

MALL

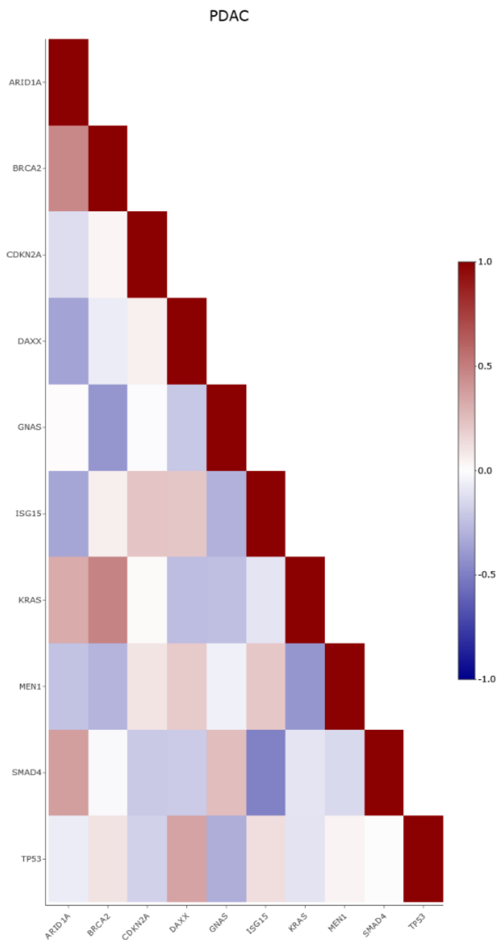

ISG15

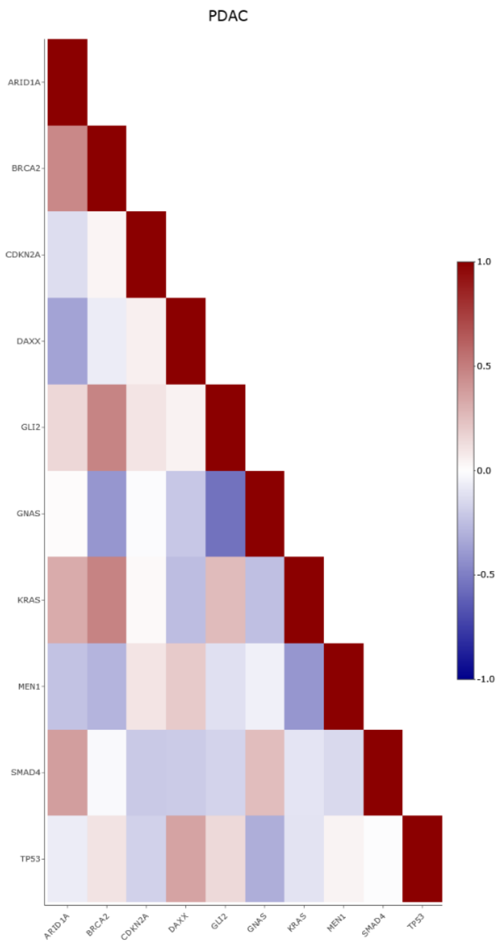

GLI2

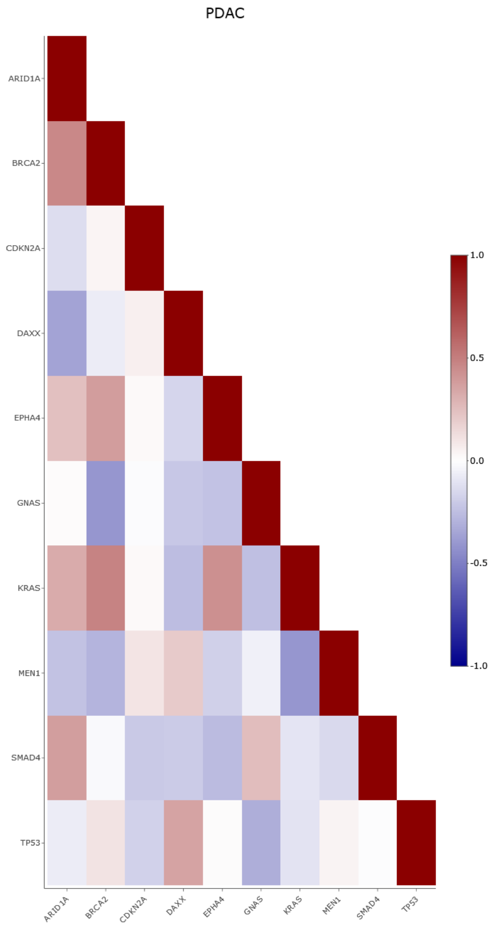

EPHA4

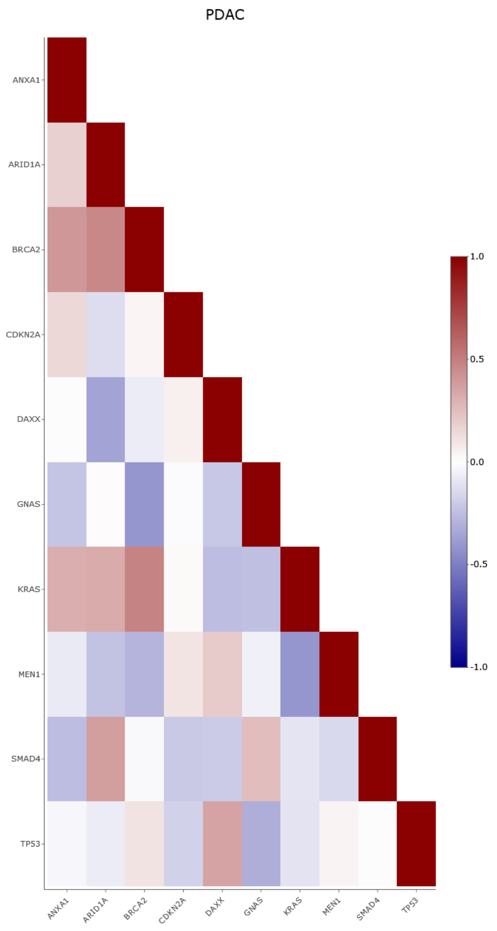

ANXA1

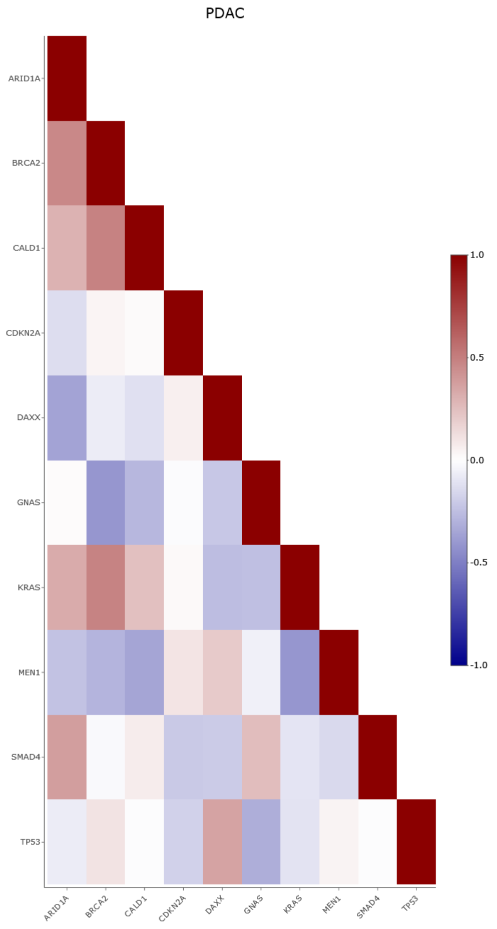

CALD1

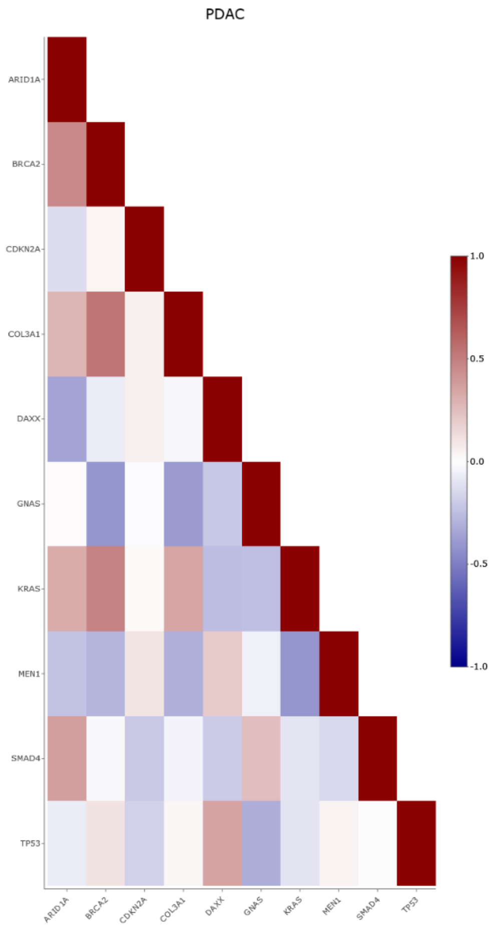

COL3A1

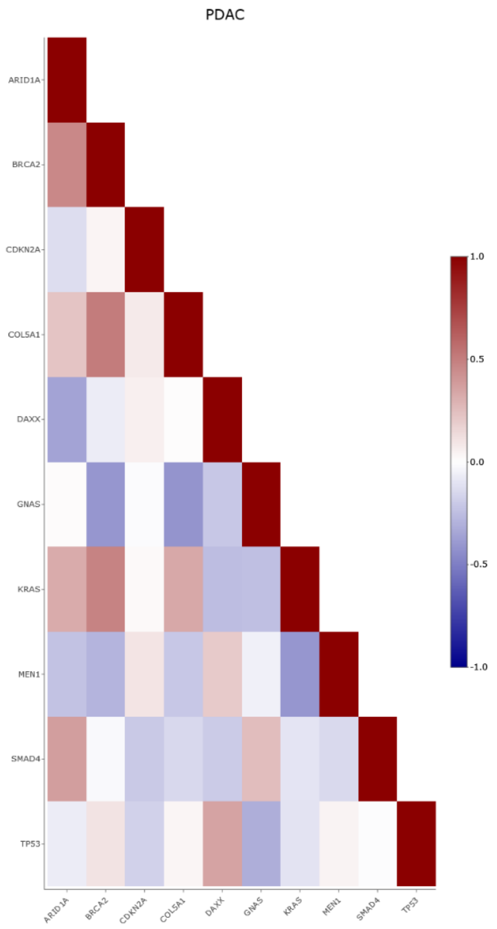

COL5A1

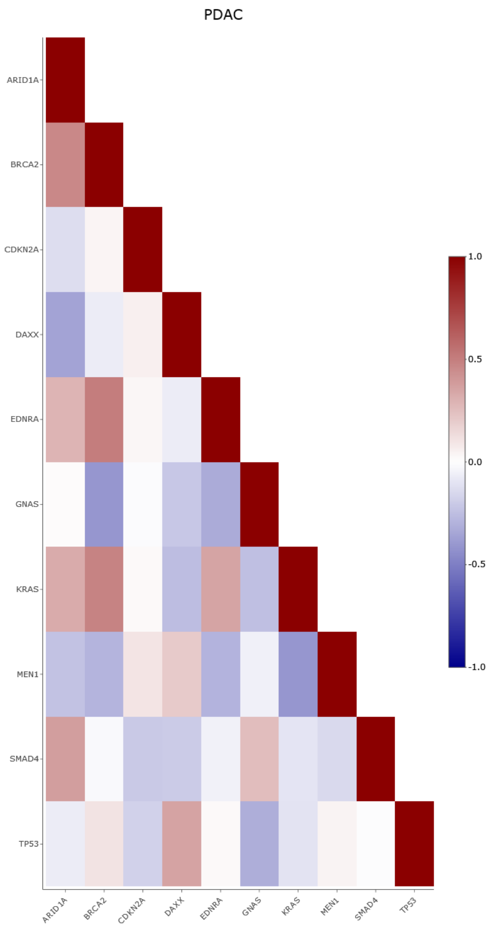

EDNRA

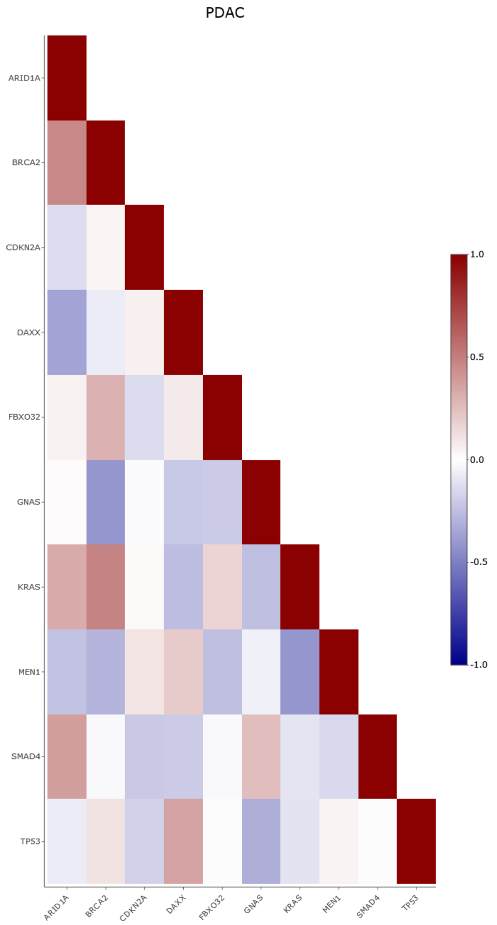

FBXO32

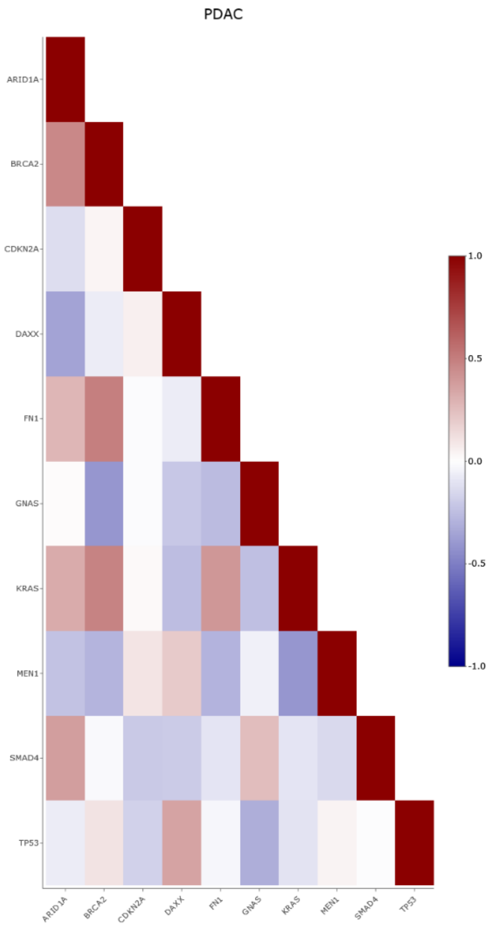

FN1

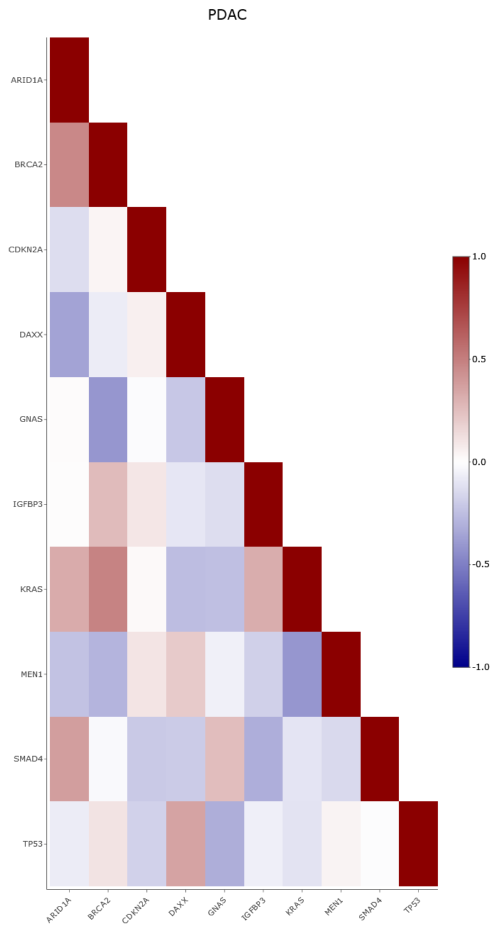

IGFBP3

b.

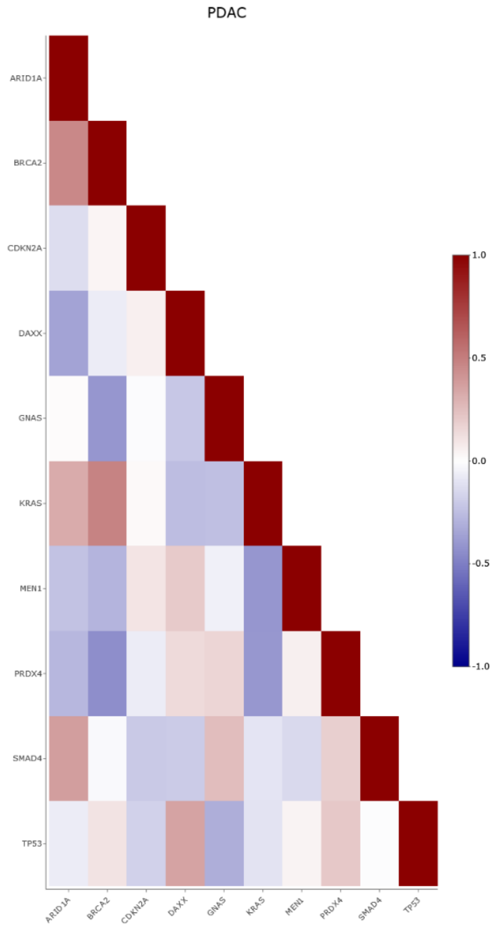

PRDX4

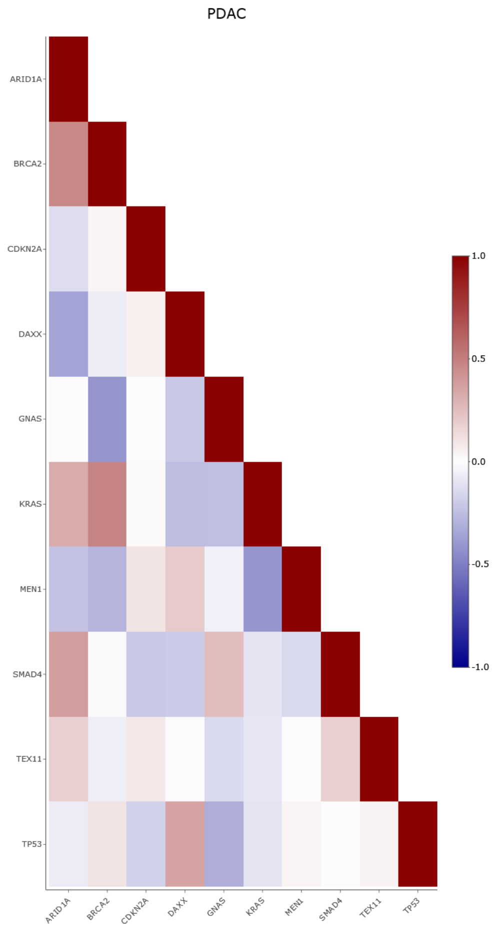

TEX11

Supplementary Figure 2. Correlation of the expression profile of identified DEGs in male PC patients with several mutated genes in PC. a. Correlation of upregulated ARE-containing genes in male patients with several mutated genes in PC among the male PC patients from TCGA data source. b. Correlation of *PRDX4* and *TEX11*, two downregulated genes in male PC patients which are located on X chromosome, with several mutated genes in PC among male PC patients from TCGA data source. The data is obtained from Pancreatic Expression Database (PED) (https://www.pancreasexpression.org/).
