# Supplementary material for: Gender-related differentially expressed genes in pancreatic cancer: possible culprits or accomplices?
Source: Front Genet. 2022 Oct 26;13:966941. doi: 10.3389/fgene.2022.966941 (PMC9643577; doi:10.3389/fgene.2022.966941)
Supplement: Supplementary file 7 [file DataSheet3.PDF]

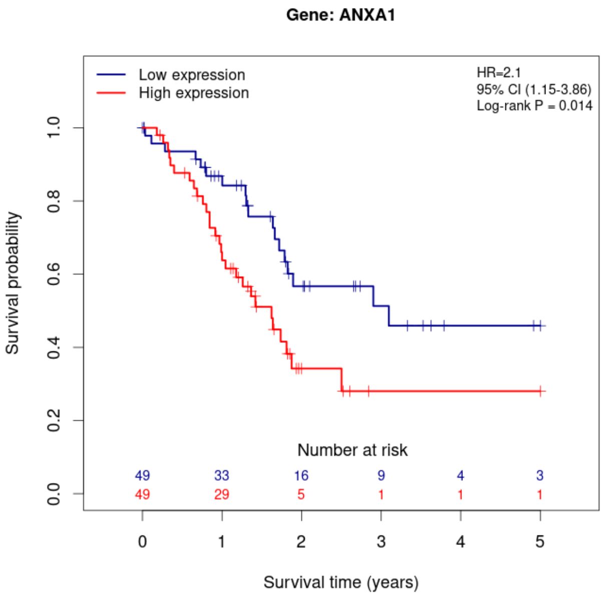
Gene: ANXA1
Low expression
High expression
HR=2.1
95% CI (1.15-3.86)
Log-rank P = 0.014
Number at risk
49 33 16 9 4 3
49 29 5 1 1 1
Survival probability
Survival time (years)

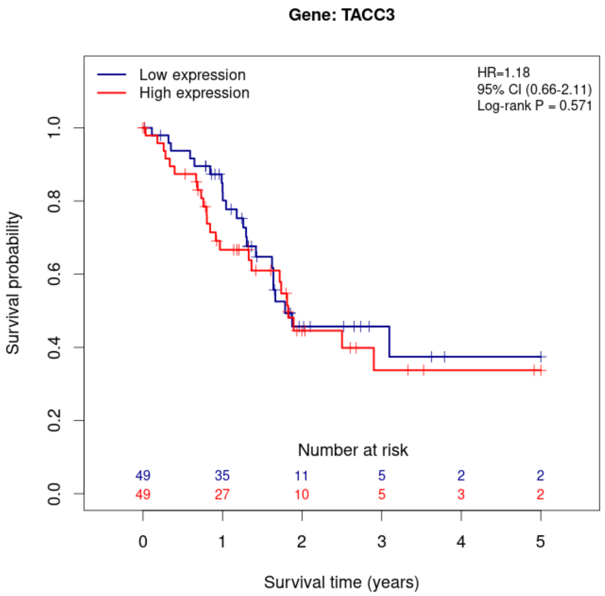
Gene: TACC3
Low expression
High expression
HR=1.18
95% CI (0.66-2.11)
Log-rank P = 0.571
Number at risk
49 35 11 5 2 2
49 27 10 5 3 2
Survival probability
Survival time (years)

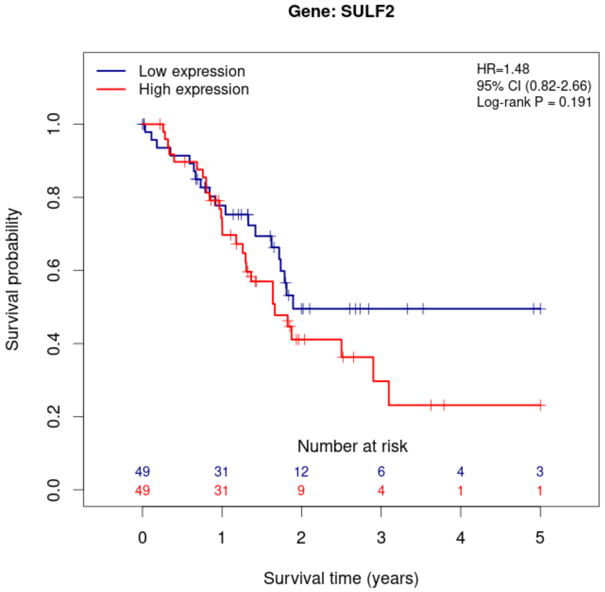
Gene: SULF2
Low expression
High expression
HR=1.48
95% CI (0.82-2.66)
Log-rank P = 0.191
Number at risk
49 31 12 6 4 3
49 31 9 4 1 1
Survival probability
Survival time (years)

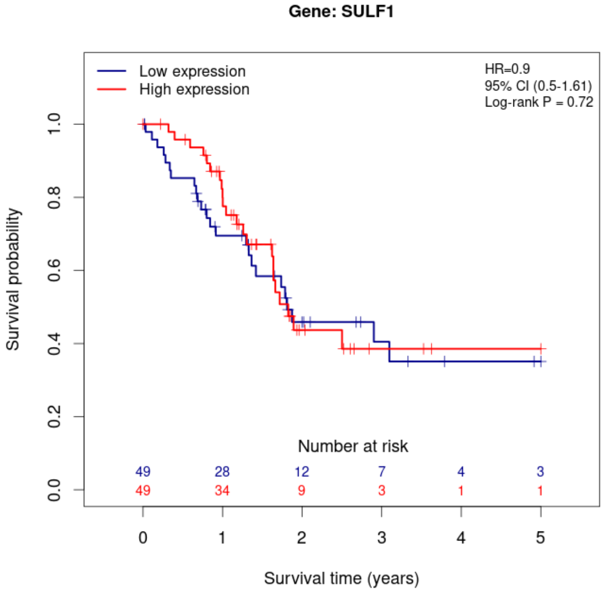
Gene: SULF1
Low expression
High expression
HR=0.9
95% CI (0.5-1.61)
Log-rank P = 0.72
Number at risk
49 28 12 7 4 3
49 34 9 3 1 1
Survival probability
Survival time (years)

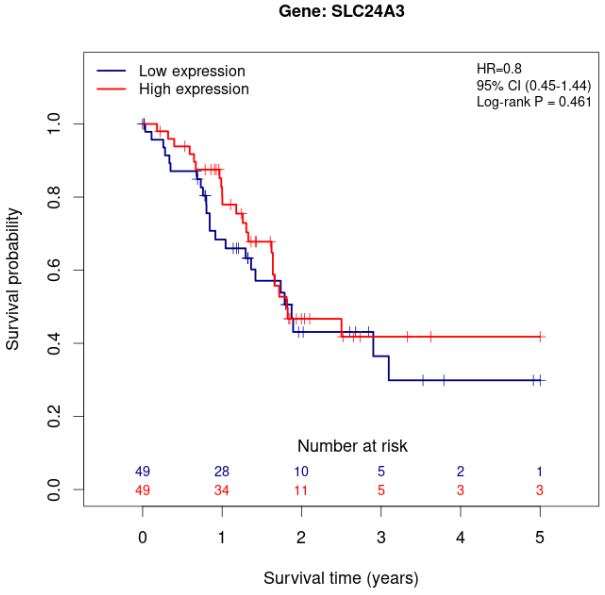
Gene: SLC24A3
Low expression
High expression
HR=0.8
95% CI (0.45-1.44)
Log-rank P = 0.461
Number at risk
49 28 10 5 2 1
49 34 11 5 3 3
Survival probability
Survival time (years)

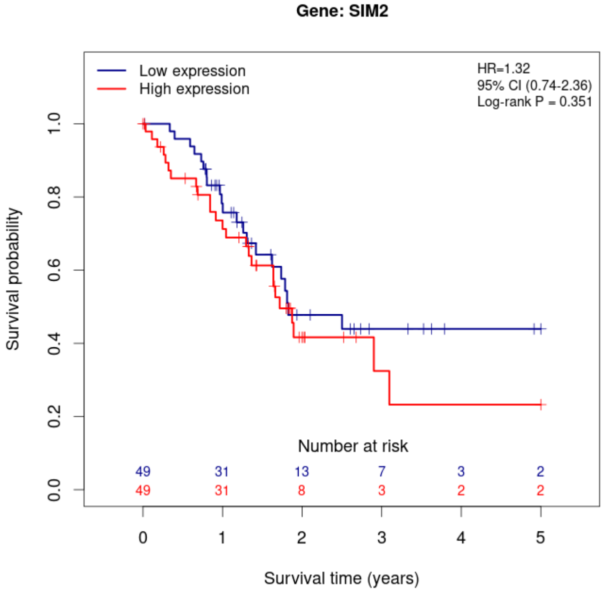
Gene: SIM2
Low expression
High expression
HR=1.32
95% CI (0.74-2.36)
Log-rank P = 0.351
Number at risk
49 31 13 7 3 2
49 31 8 3 2 2
Survival probability
Survival time (years)

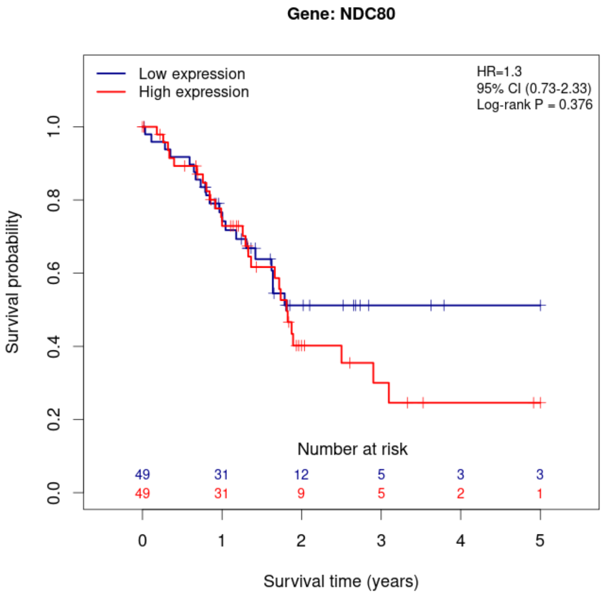
Gene: NDC80
Low expression
High expression
HR=1.3
95% CI (0.73-2.33)
Log-rank P = 0.376
Survival probability
Number at risk
49 31 12 5 3 3
49 31 9 5 2 1
Survival time (years)

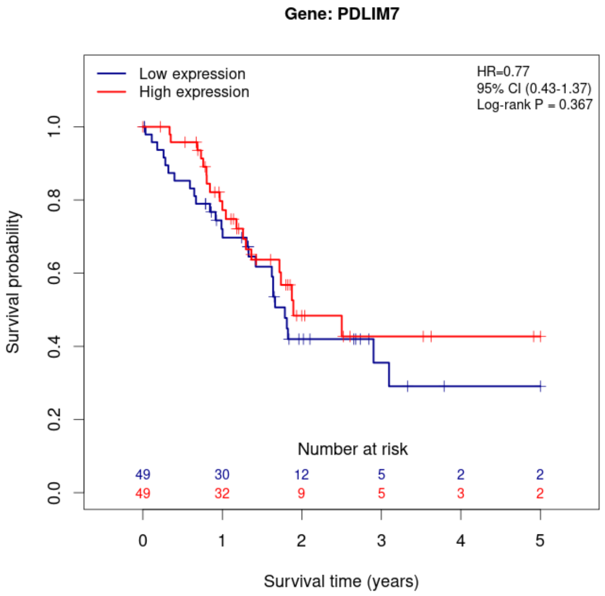
Gene: PDLIM7
Low expression
High expression
HR=0.77
95% CI (0.43-1.37)
Log-rank P = 0.367
Survival probability
Number at risk
49 30 12 5 2 2
49 32 9 5 3 2
Survival time (years)

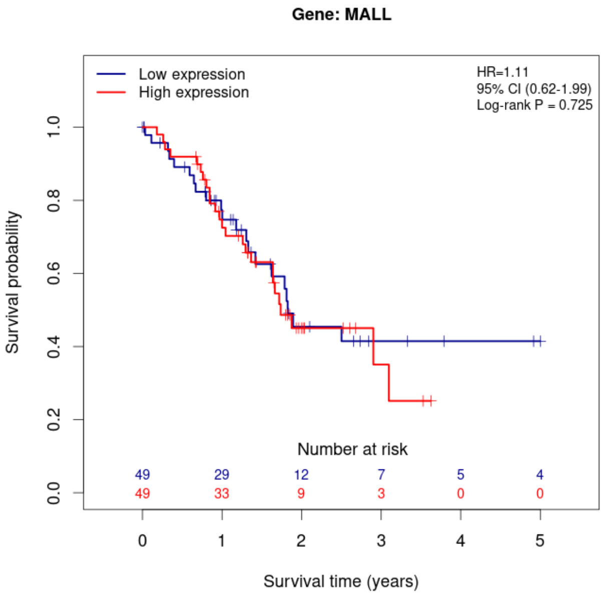
Gene: MALL
Low expression
High expression
HR=1.11
95% CI (0.62-1.99)
Log-rank P = 0.725
Survival probability
Number at risk
49 29 12 7 5 4
49 33 9 3 0 0
Survival time (years)

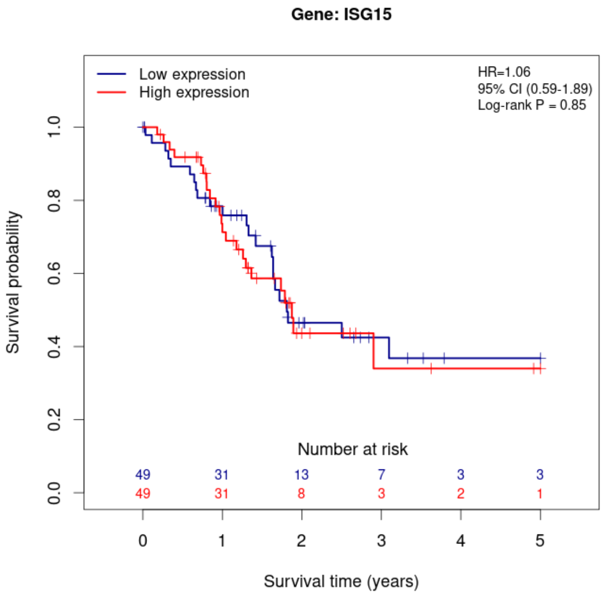
Gene: ISG15
Low expression
High expression
HR=1.06
95% CI (0.59-1.89)
Log-rank P = 0.85
Survival probability
Number at risk
49 31 13 7 3 3
49 31 8 3 2 1
Survival time (years)

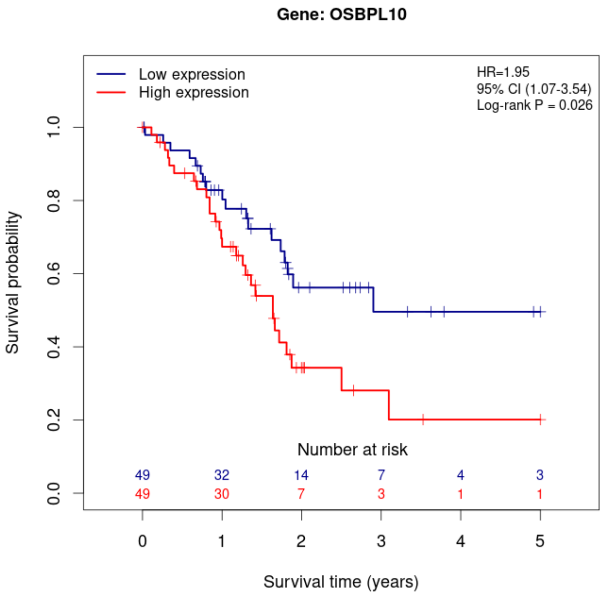
Gene: OSBPL10
Low expression
High expression
HR=1.95
95% CI (1.07-3.54)
Log-rank P = 0.026
Survival probability
Number at risk
49 32 14 7 4 3
49 30 7 3 1 1
Survival time (years)

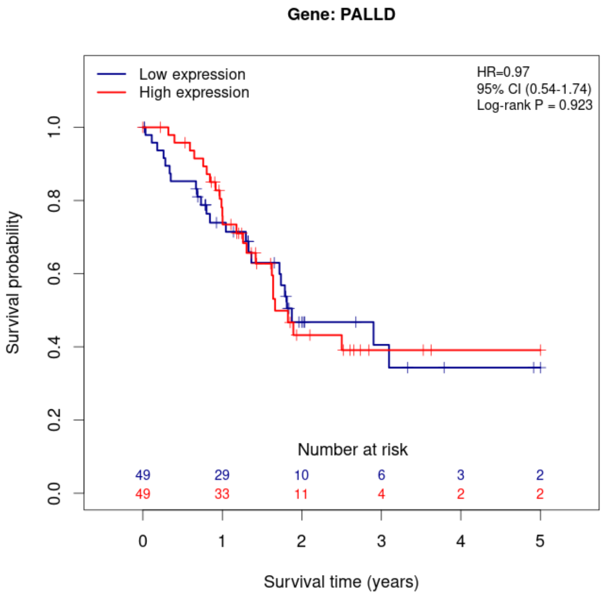
Gene: PALLD
Low expression
High expression
HR=0.97
95% CI (0.54-1.74)
Log-rank P = 0.923
Survival probability
Number at risk
49 29 10 6 3 2
49 33 11 4 2 2
Survival time (years)

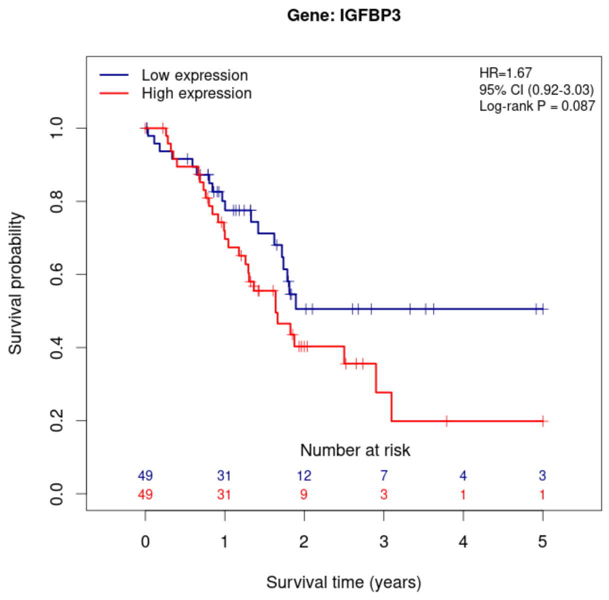
Gene: IGFBP3
Low expression
High expression
HR=1.67
95% CI (0.92-3.03)
Log-rank P = 0.087
Survival probability
Number at risk
49 31 12 7 4 3
49 31 9 3 1 1
Survival time (years)

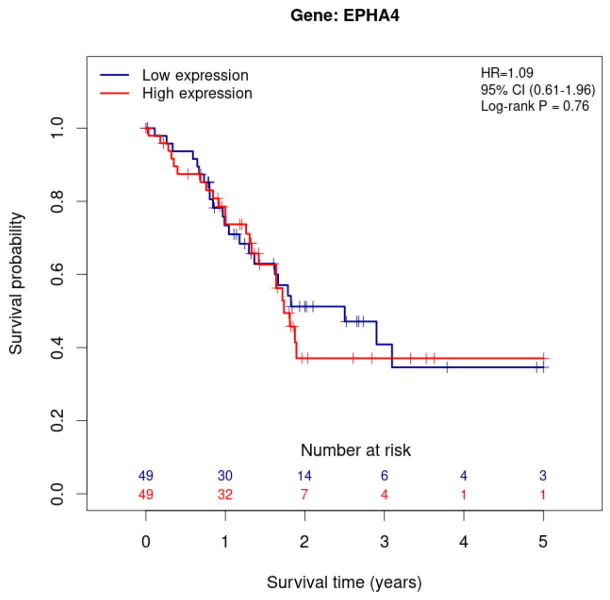
Gene: EPHA4
Low expression
High expression
HR=1.09
95% CI (0.61-1.96)
Log-rank P = 0.76
Survival probability
Number at risk
49 30 14 6 4 3
49 32 7 4 1 1
Survival time (years)

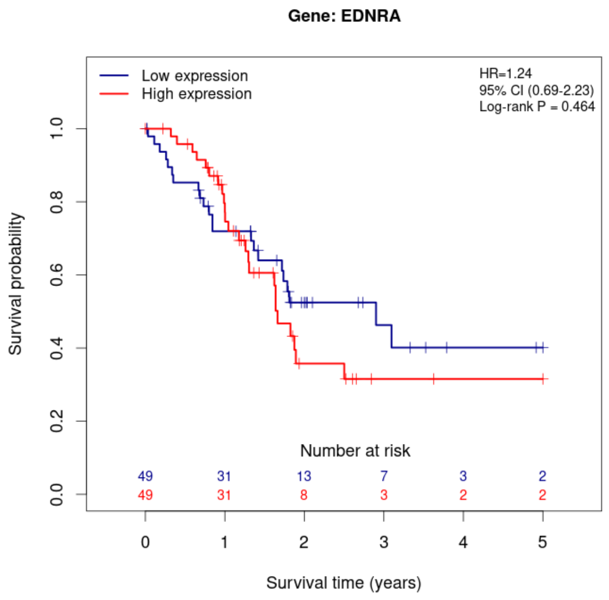
Gene: EDNRA
Low expression
High expression
HR=1.24
95% CI (0.69-2.23)
Log-rank P = 0.464
Survival probability
Number at risk
49 31 13 7 3 2
49 31 8 3 2 2
Survival time (years)

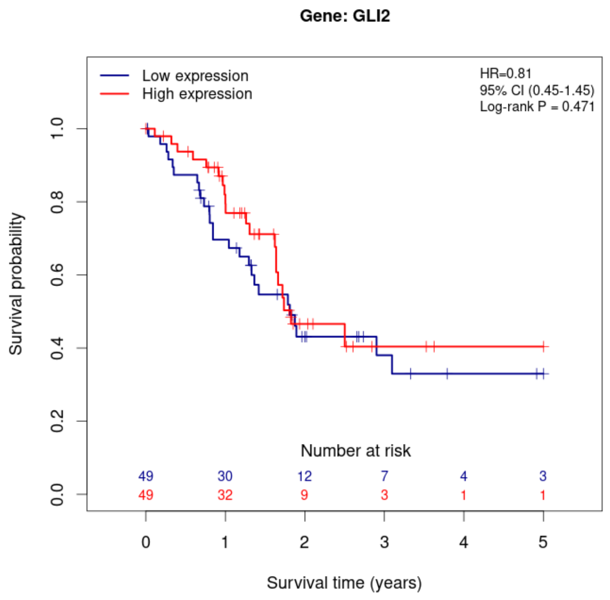
Gene: GLI2
Low expression
High expression
HR=0.81
95% CI (0.45-1.45)
Log-rank P = 0.471
Survival probability
Number at risk
49 30 12 7 4 3
49 32 9 3 1 1
Survival time (years)

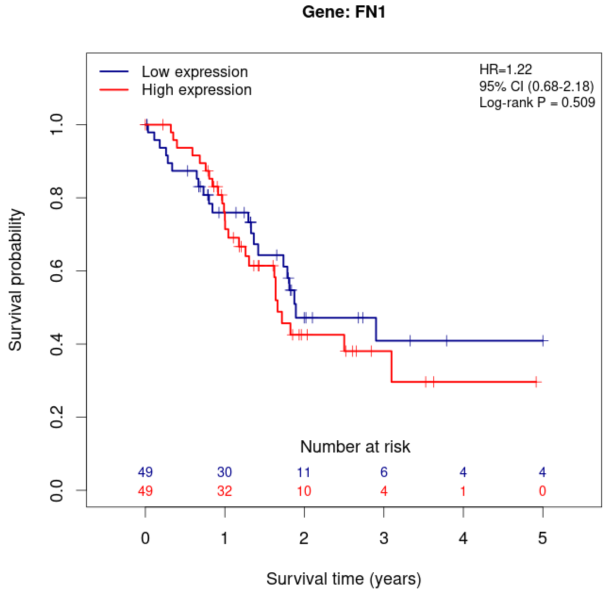
Gene: FN1
Low expression
High expression
HR=1.22
95% CI (0.68-2.18)
Log-rank P = 0.509
Survival probability
Number at risk
49 30 11 6 4 4
49 32 10 4 1 0
Survival time (years)

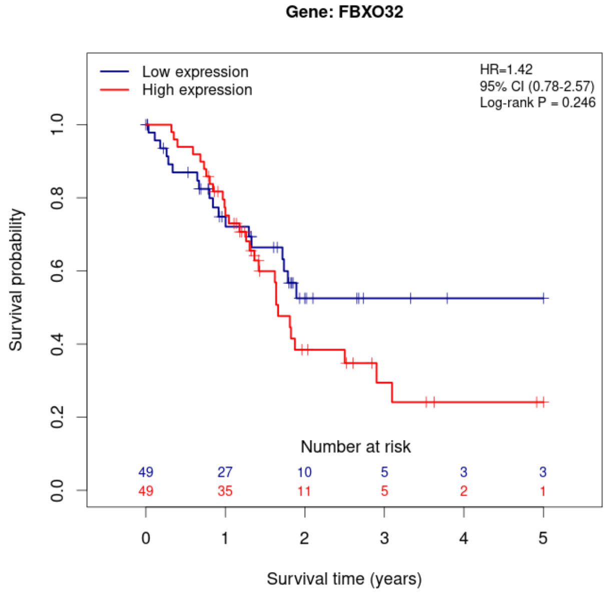
Gene: FBXO32
Low expression
High expression
HR=1.42
95% CI (0.78-2.57)
Log-rank P = 0.246
Survival probability
Number at risk
49 27 10 5 3 3
49 35 11 5 2 1
Survival time (years)

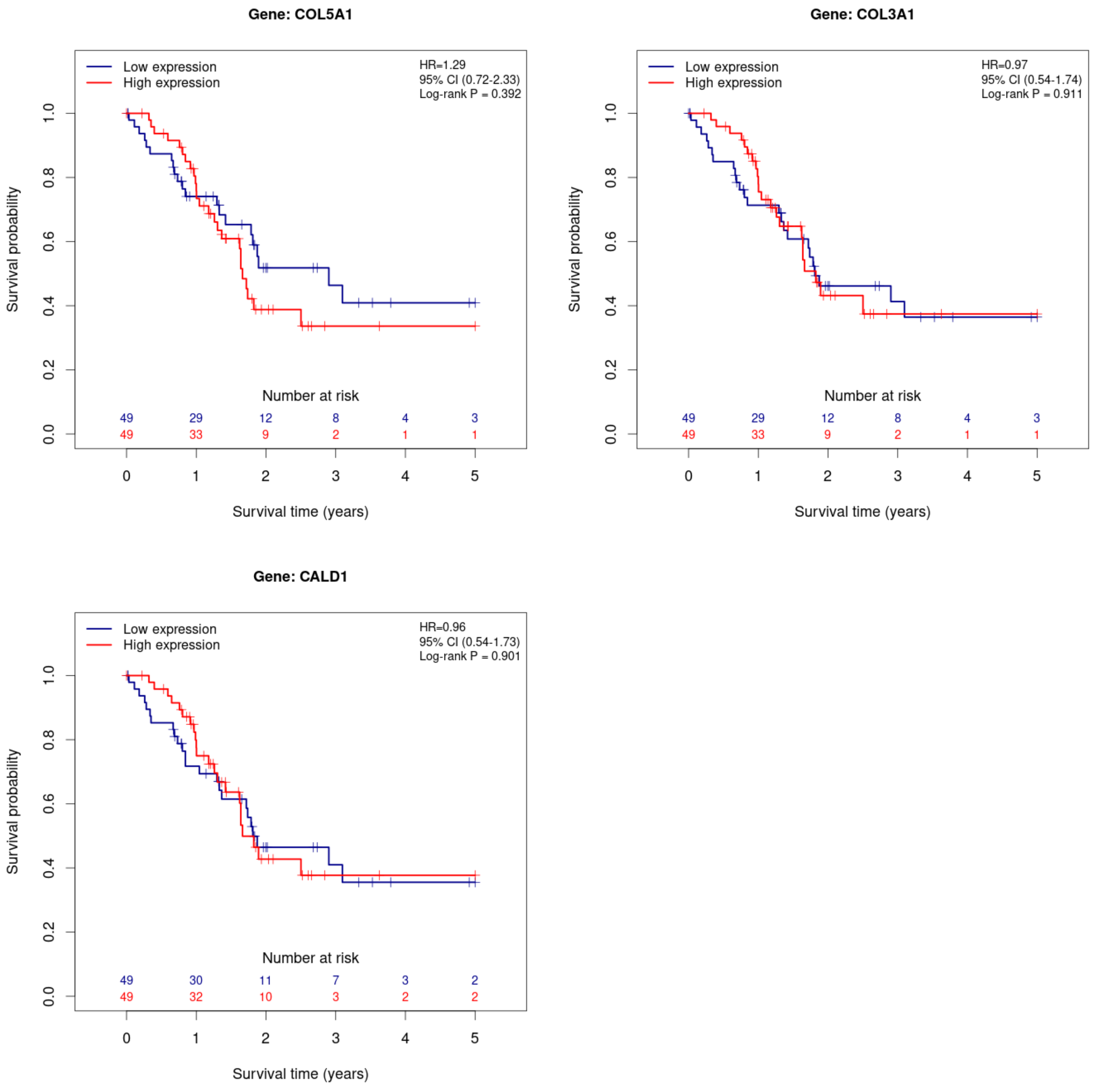

Supplementary Figure 3. Survival analysis of upregulated ARE-containing genes in male patients, in male samples of PC from TCGA data source. The data is obtained from Pancreatic Expression Database (PED) (https://www.pancreasexpression.org/).
