# Supplementary material for: Gender-related differentially expressed genes in pancreatic cancer: possible culprits or accomplices?
Source: Front Genet. 2022 Oct 26;13:966941. doi: 10.3389/fgene.2022.966941 (PMC9643577; doi:10.3389/fgene.2022.966941)
Supplement: Supplementary file 9 [file Image3.pdf]

Gene: ANXA1

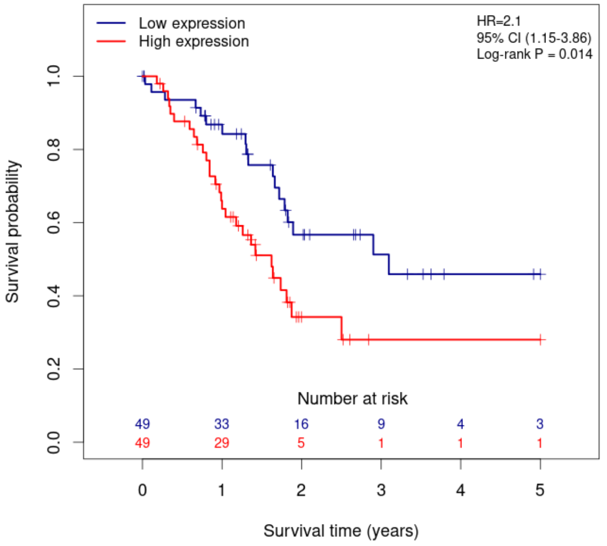

Gene: TACC3

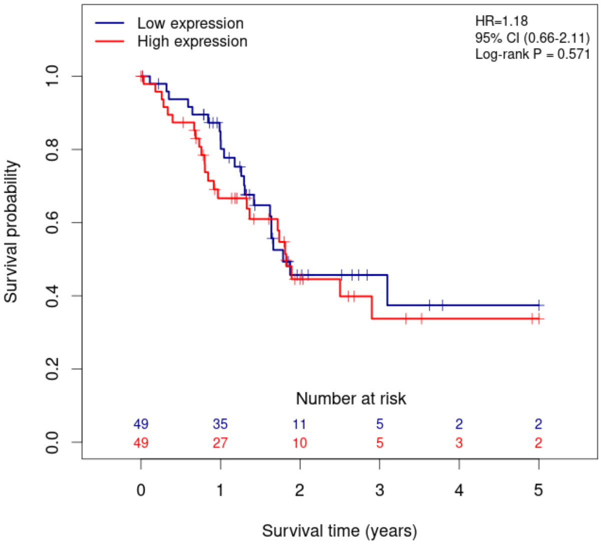

Gene: SULF2

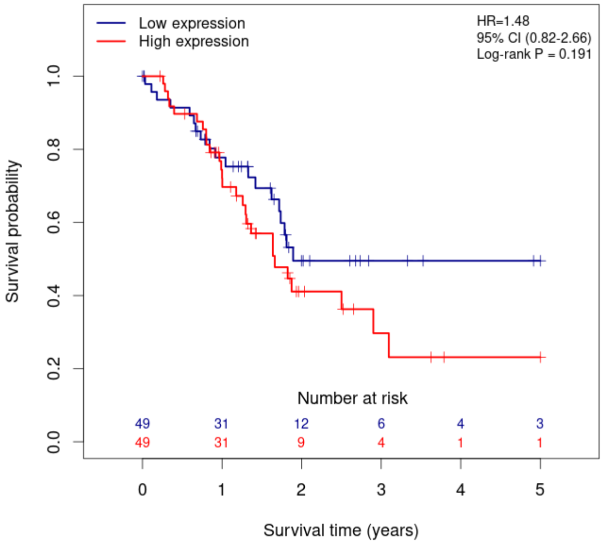

Gene: SULF1

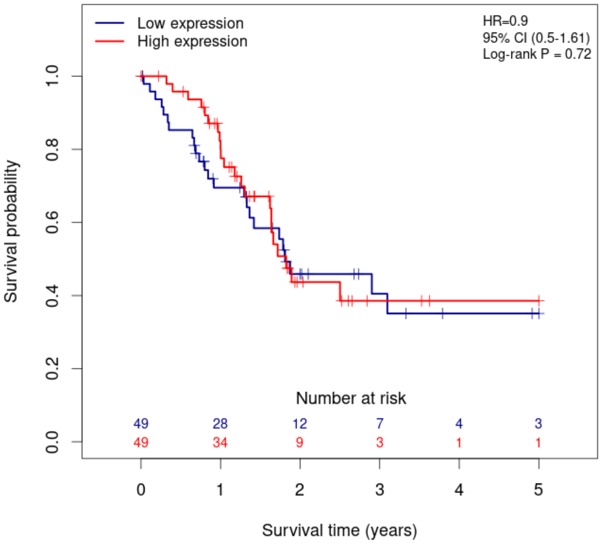

Gene: SLC24A3

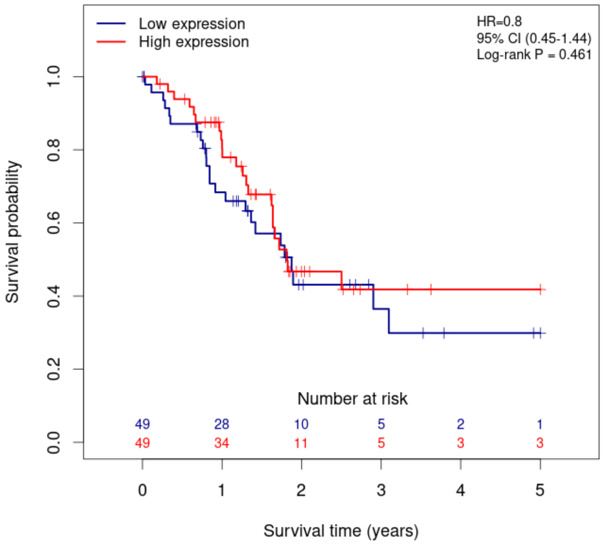

Gene: SIM2

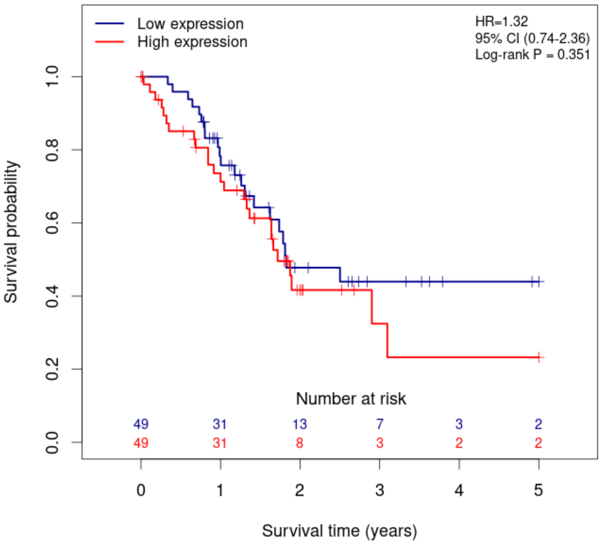

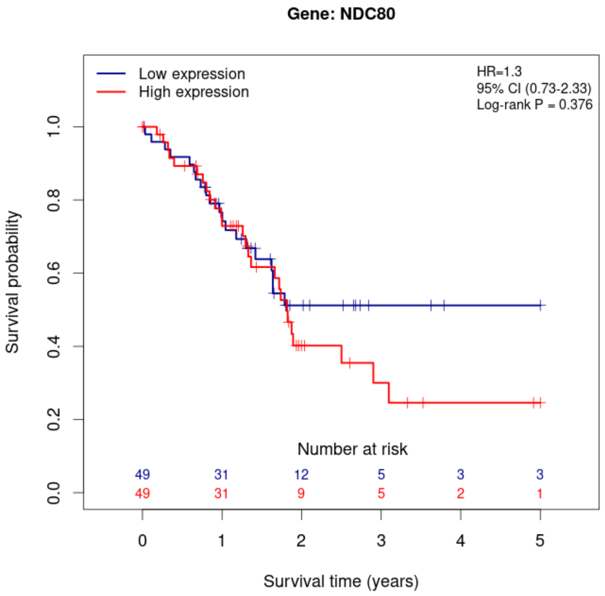
Gene: NDC80
Low expression
High expression
HR=1.3
95% CI (0.73-2.33)
Log-rank P = 0.376
Survival probability
0.0 0.2 0.4 0.6 0.8 1.0
Number at risk
49 31 12 5 3 3
49 31 9 5 2 1
0 1 2 3 4 5
Survival time (years)

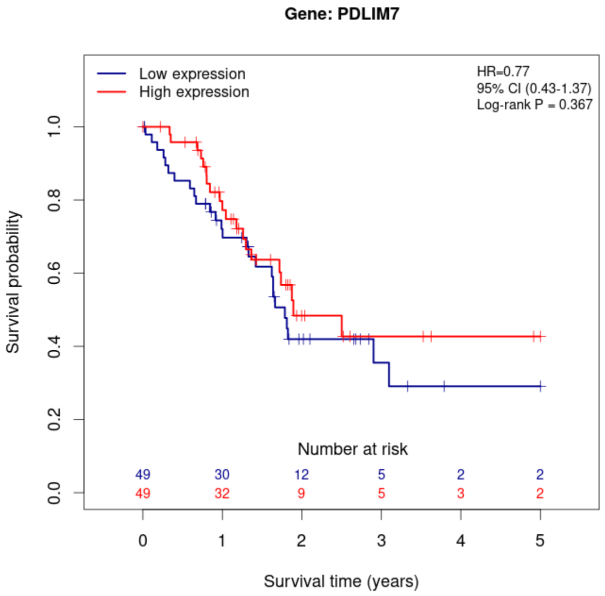
Gene: PDLIM7
Low expression
High expression
HR=0.77
95% CI (0.43-1.37)
Log-rank P = 0.367
Survival probability
0.0 0.2 0.4 0.6 0.8 1.0
Number at risk
49 30 12 5 2 2
49 32 9 5 3 2
0 1 2 3 4 5
Survival time (years)

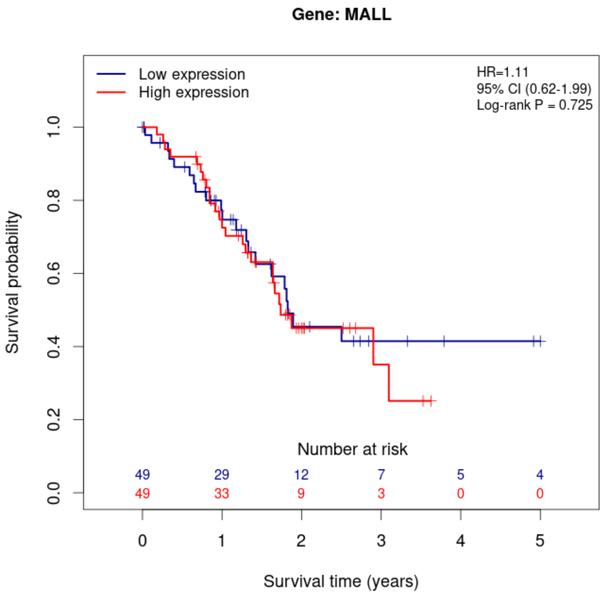
Gene: MALL
Low expression
High expression
HR=1.11
95% CI (0.62-1.99)
Log-rank P = 0.725
Survival probability
0.0 0.2 0.4 0.6 0.8 1.0
Number at risk
49 29 12 7 5 4
49 33 9 3 0 0
0 1 2 3 4 5
Survival time (years)

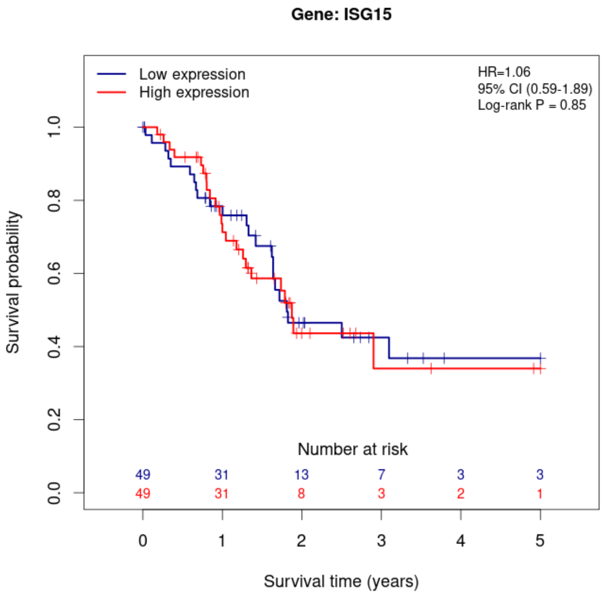
Gene: ISG15
Low expression
High expression
HR=1.06
95% CI (0.59-1.89)
Log-rank P = 0.85
Survival probability
0.0 0.2 0.4 0.6 0.8 1.0
Number at risk
49 31 13 7 3 3
49 31 8 3 2 1
0 1 2 3 4 5
Survival time (years)

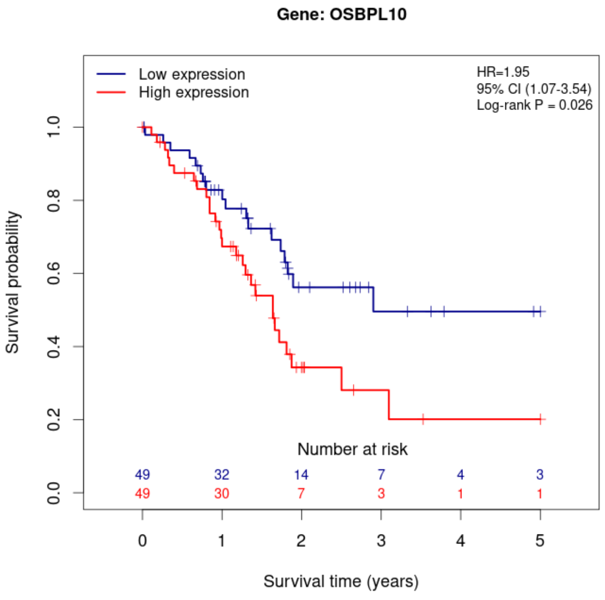
Gene: OSBPL10
Low expression
High expression
HR=1.95
95% CI (1.07-3.54)
Log-rank P = 0.026
Survival probability
0.0 0.2 0.4 0.6 0.8 1.0
Number at risk
49 32 14 7 4 3
49 30 7 3 1 1
0 1 2 3 4 5
Survival time (years)

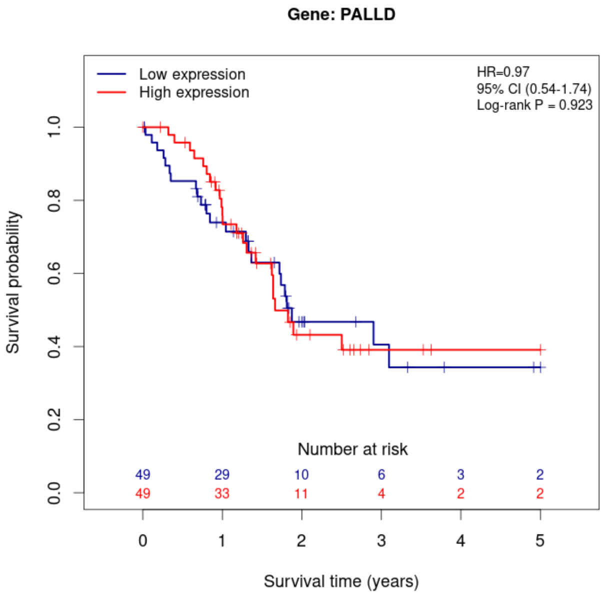
Gene: PALLD
Low expression
High expression
HR=0.97
95% CI (0.54-1.74)
Log-rank P = 0.923
Survival probability
0.0 0.2 0.4 0.6 0.8 1.0
Number at risk
49 29 10 6 3 2
49 33 11 4 2 2
0 1 2 3 4 5
Survival time (years)

Gene: IGFBP3

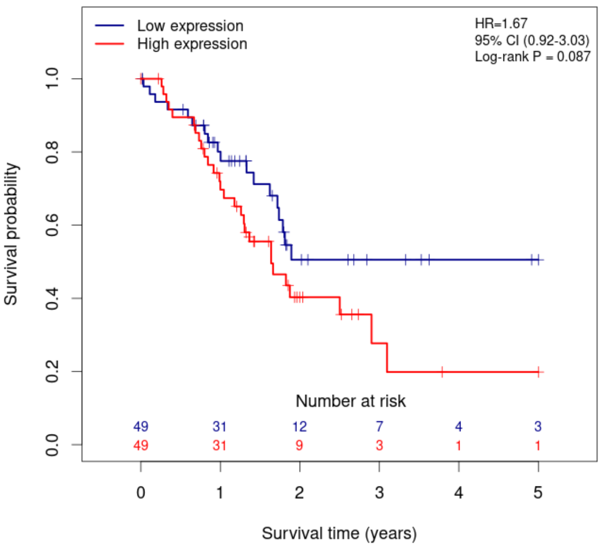

Gene: EPHA4

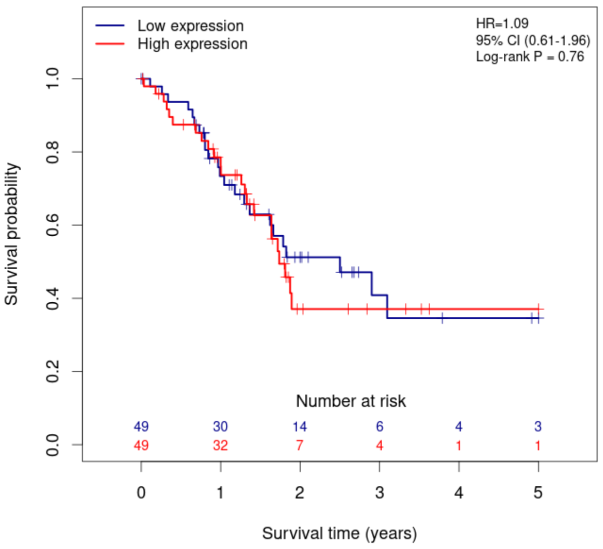

Gene: EDNRA

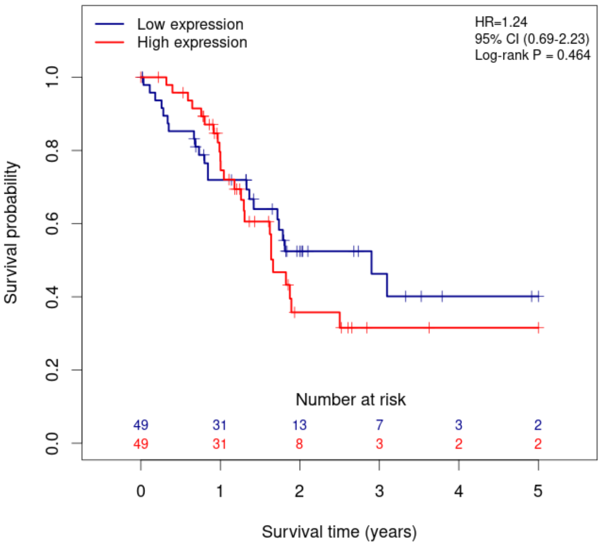

Gene: GLI2

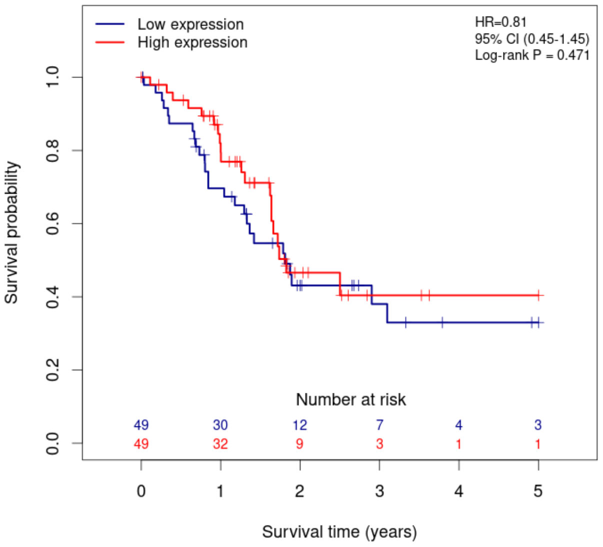

Gene: FN1

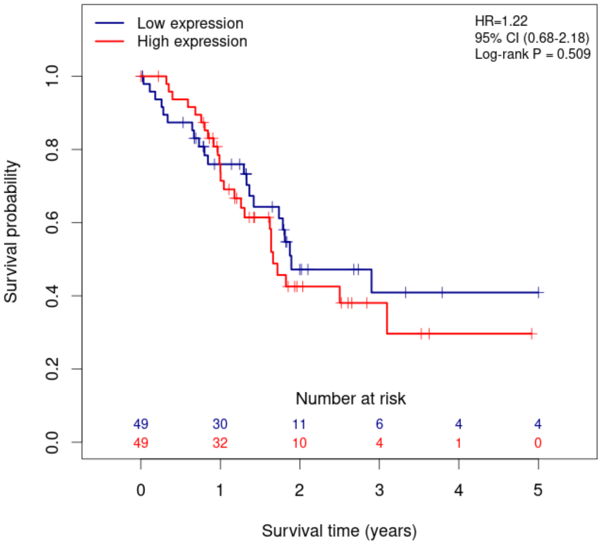

Gene: FBXO32

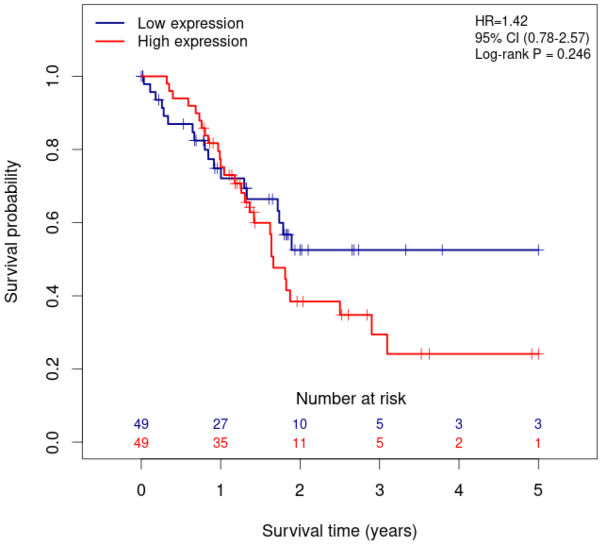

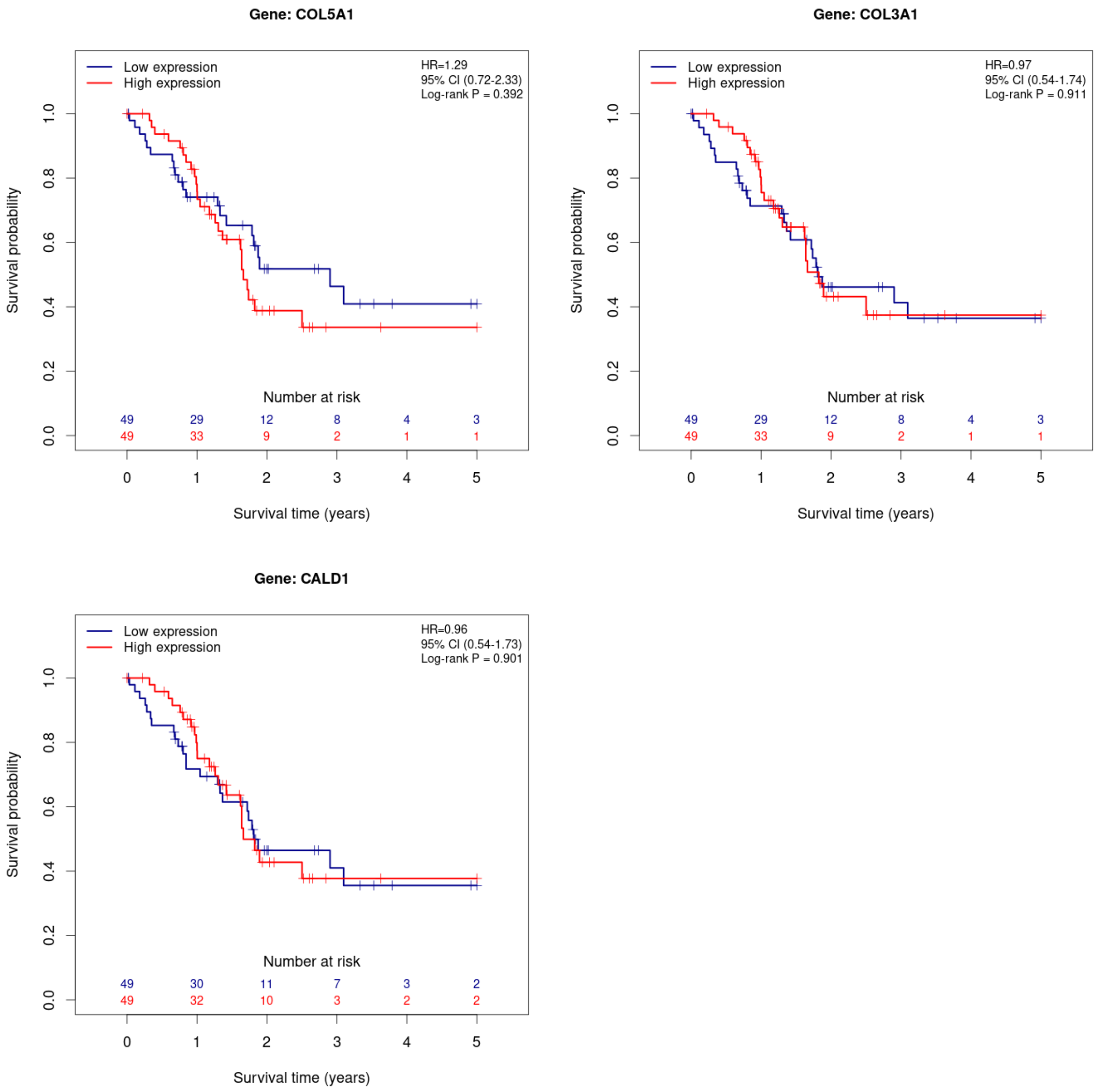

Supplementary Figure 3. Survival analysis of upregulated ARE-containing genes in male patients, among male PC patients from TCGA data source. The data is obtained from Pancreatic Expression Database (PED) (https://www.pancreasexpression.org/).
