# Supplementary material for: Gender-related differentially expressed genes in pancreatic cancer: possible culprits or accomplices?
Source: Front Genet. 2022 Oct 26;13:966941. doi: 10.3389/fgene.2022.966941 (PMC9643577; doi:10.3389/fgene.2022.966941)
Supplement: Supplementary file 10 [file DataSheet1.PDF]

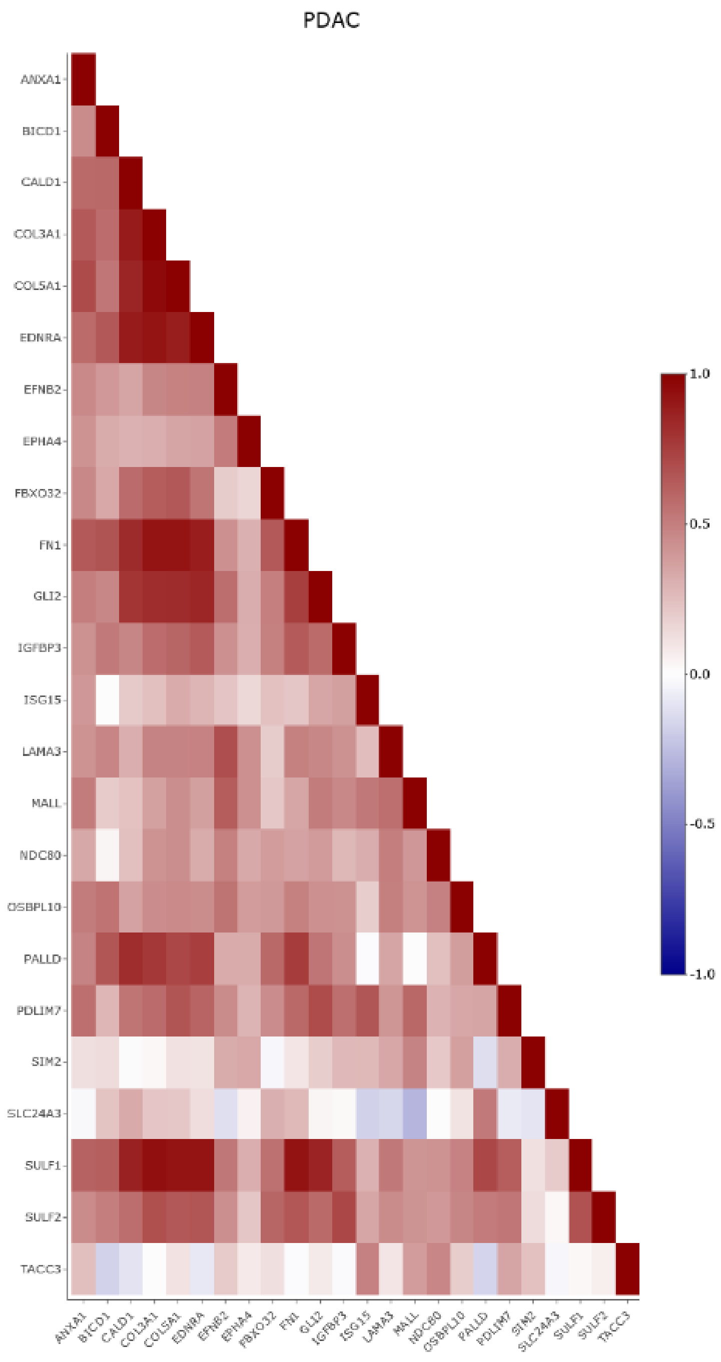

(Male patients according to TCGA)

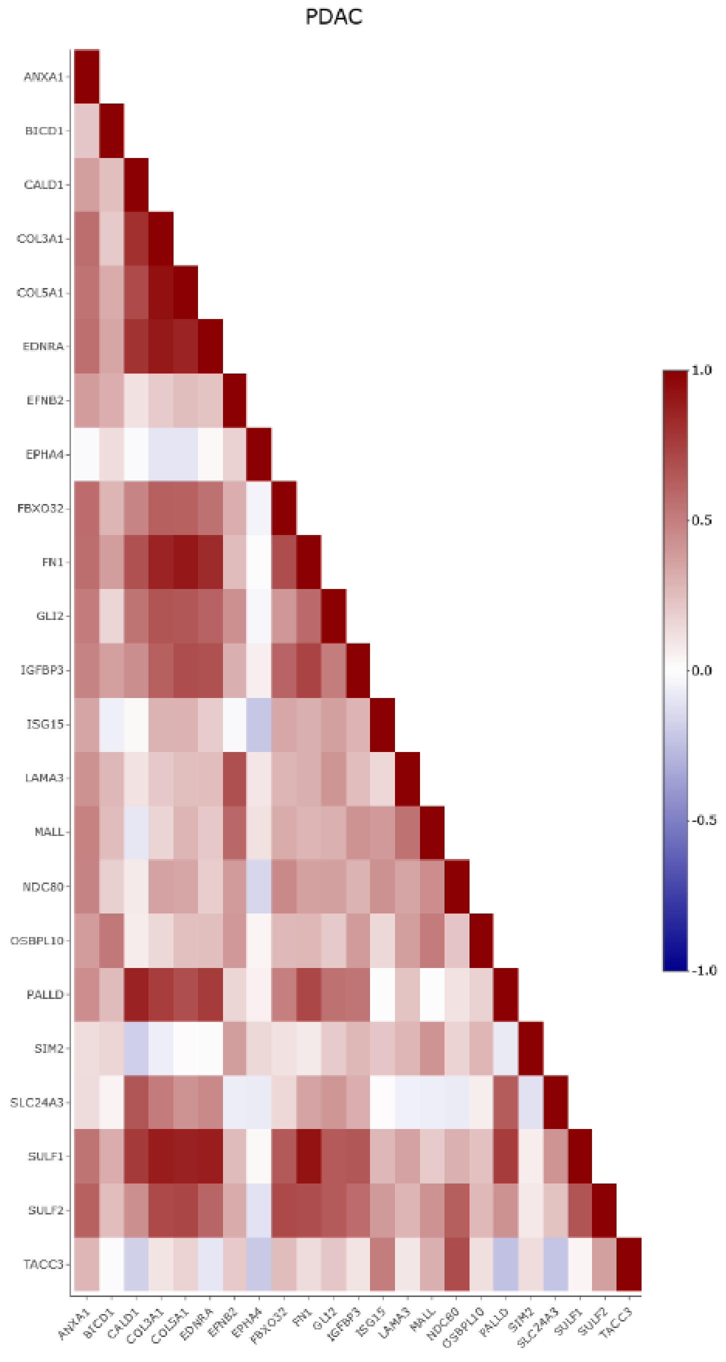

(Female patients according to TCGA)

Supplementary figure 1. ARE-containing genes which were upregulated in male patients seem to have a higher correlation in their expression profiles with each other, in male patients in comparison with female PC patients from the TCGA data source, based on Pearson Product Moment Correlation Coefficient (PMCC) value.
